# Therapeutic FGF19 promotes HDL biogenesis and transhepatic cholesterol efflux to prevent atherosclerosis[S]

**DOI:** 10.1194/jlr.M089961

**Published:** 2019-01-24

**Authors:** Mei Zhou, R. Marc Learned, Stephen J. Rossi, Hui Tian, Alex M. DePaoli, Lei Ling

**Affiliations:** NGM Biopharmaceuticals, Inc., South San Francisco, CA 94080

**Keywords:** adenosine 5′-triphosphate-binding cassette transporter A1, bile acid metabolism, cholesterol 7-alpha hydroxylase, clinical studies, liver, nuclear receptor liver X receptor, nonalcoholic fatty liver disease, steatohepatitis, fibroblast growth factor 19, high density lipoprotein

## Abstract

Fibroblast growth factor (FGF)19, an endocrine hormone produced in the gut, acts in the liver to control bile acid synthesis. NGM282, an engineered FGF19 analog, is currently in clinical development for treating nonalcoholic steatohepatitis. However, the molecular mechanisms that integrate FGF19 with cholesterol metabolic pathways are incompletely understood. Here, we report that FGF19 and NGM282 promote HDL biogenesis and cholesterol efflux from the liver by selectively modulating LXR signaling while ameliorating hepatic steatosis. We further identify ABCA1 and FGF receptor 4 as mediators of this effect, and that administration of a HMG-CoA reductase inhibitor or a blocking antibody against proprotein convertase subtilisin/kexin type 9 abolished FGF19-associated elevations in total cholesterol, HDL cholesterol (HDL-C), and LDL cholesterol in *db/db* mice. Moreover, we show that a constitutively active MEK1, but not a constitutively active STAT3, mimics the effect of FGF19 and NGM282 on cholesterol change. In dyslipidemic *Apoe*^−/−^ mice fed a Western diet, treatment with NGM282 dramatically reduced atherosclerotic lesion area in aortas. Administration of NGM282 to healthy volunteers for 7 days resulted in a 26% increase in HDL-C levels compared with placebo. These findings outline a previously unrecognized role for FGF19 in the homeostatic control of cholesterol and may have direct impact on the clinical development of FGF19 analogs.

Homeostasis of cholesterol, an important risk factor for coronary artery disease, is maintained by the coordinated regulation of de novo synthesis, catabolism, nutritional intake, intestinal absorption, reverse transport, and biliary excretion (1). Genome-wide association studies have uncovered a plethora of genetic loci associated with alterations in plasma cholesterol levels that have provided valuable mechanistic insights (2). Despite notable successes in developing medical therapies to reduce plasma levels of LDL cholesterol (LDL-C), cardiovascular disease remains the leading cause of death and years-of-life-lost worldwide (3). This high unmet medical need necessitates going beyond the established paradigm to explore alternative approaches to lower the risk of cardiovascular disease, especially those of an atherosclerotic nature.

Plasma HDL cholesterol (HDL-C) concentrations inversely associate with the risk of cardiovascular disease (4), a finding that has led to the hypothesis that HDL-C may protect against cardiovascular disease. Indeed, the development of novel therapies to exploit the atheroprotective property of HDL has been an area of intense investigation in recent years, but has proven to be particularly challenging, as high-profile clinical trials of drugs targeting the cholesteryl ester transfer protein ended in disappointment (5, 6).

Fibroblast growth factor (FGF)19 is an endocrine hormone that has a crucial role in controlling bile acid, carbohydrate, protein, and energy homeostasis (7, 8). Through the FGF receptor (FGFR)4-βKlotho receptor complex, FGF19 potently suppresses mRNA levels of *CYP7A1*, the gene encoding cholesterol-7α-hydroxylase, to inhibit de novo bile acid synthesis (7, 8). FGF19 also increases metabolic rate and reduces adiposity (9), acts as a postprandial insulin-independent activator of hepatic glycogen and protein synthesis (10), and regulates hepatic glucose metabolism by inhibiting the CREB-PGC1α pathway (11). The effect on weight reduction and glycemic regulation by FGF19 is largely dependent on the FGFR1c-βKlotho receptor complex, through its actions on the nervous system (12–14). In recent years, FGF19 has emerged as an attractive target for treating chronic liver diseases where bile acids play pivotal roles, and to this end, an engineered nontumorigenic analog of FGF19, NGM282 (also known as M70) (15), is currently being evaluated as a potential treatment for patients with nonalcoholic steatohepatitis, primary sclerosing cholangitis, or primary biliary cholangitis (16, 17). In NGM282, the deletion of five amino acids (P24–S28) coupled with the substitution of three amino acids at critical positions (A30S, G31S, H33L) within the N terminus enables biased FGFR4 signaling so that NGM282 retains the ability to repress CYP7A1 expression, but is unable to activate signal transducer and activator of transcription 3 (STAT3) signaling to trigger hepatic tumorigenesis (15, 18).

In this study, we describe a previously unrecognized role of FGF19 in selectively modulating hepatic LXR signaling to promote transhepatic cholesterol efflux and to increase HDL biogenesis, and that the effects of FGF19 on cholesterol metabolism include a complex interplay between multiple regulatory pathways not limited to the inhibition of bile acid synthesis. We also evaluated the effects of an FGF19 analog on aortic plaque and lesion formation in mouse models of atherosclerosis and on plasma cholesterol levels in healthy human volunteers.

## MATERIALS AND METHODS

### Animal studies

All animal experiments were approved by the Institutional Animal Care and Use Committee at NGM and conducted in compliance with ethical regulations. Mice were housed in a pathogen-free animal facility at 22°C under controlled 12 h light and12 h dark cycles. All mice were maintained in filter-topped cages on standard chow diet (Teklad 2918), or special diets when indicated, and autoclaved water ad libitum. Male mice were used unless otherwise specified. Sample sizes were determined on the basis of prior experience in the selected models to allow detection of statistically significant differences in metabolic parameters between groups. Mice were randomized into the treatment groups based on body weight and blood glucose. Studies were replicated in two to three independent cohorts of animals. All injections and tests were performed during the light cycle. Technicians treating the animals and measuring aortic lesions were blinded to the identity of the test articles. No animals were excluded from analysis at study completion. The *db/db* mice (BKS.Cg-Dock7^m^ +/+ Lepr^db^/J, #000642), *Abca1 ^fl/fl^Abcg1 ^fl/fl^* mice (B6.Cg-Abca1^tm1Jp^ Abcg1^tm1Tall^/J, #21067), *Apoe*^−/−^ mice (B6.129P2-Apoe^tm1Unc^/J, #002052), *Ldlr*^−/−^ mice (B6.129S7-Ldlr^tm1Her^/J, #002207), and C57BL6/J mice (#000664) were obtained from Jackson Laboratory. *Fgfr4*^−/−^ mice were kindly provided by Dr. Grace Guo (Rutgers University).

The diabetic *db/db* mouse model was chosen because it allowed us to simultaneously study lipid and glucose regulations by FGF19. The *db/db* mice (10–12 weeks old) received a single 200 μl intravenous injection of 1 × 10^11^ vector genomes (vg) of adeno-associated virus (AAV)-FGF19, AAV-NGM282, or a control virus encoding green fluorescent protein via tail vein. Animals were euthanized and livers were collected 2 weeks after injection of the AAV vectors for gene expression analysis. For experiments investigating constitutively active MAPK/ERK kinase 1 (caMEK1) or constitutively active STAT3 (caSTAT3), mice were administered with AAV-caMEK1 (1 × 10^11^ vg), AAV-caSTAT3 (1 × 10^11^ vg), AAV-FGF19 (1 × 10^11^ vg), or a control virus, and blood was collected 4 weeks after AAV injection for measurements of serum levels of cholesterol, HDL-C, and LDL-C. For experiments investigating various inhibitors, *db/db* mice were administered with AAV-FGF19 (1 × 10^11^ vg) via tail vein. Three weeks later, mice were treated with rosuvastatin (0.005% in diet; BioServ), ezetimibe (0.01% in diet; BioServ), or anti-proprotein convertase subtilisin/kexin type 9 (PCSK9) neutralizing antibody (10 mg kg^−1^ ip qw) for an additional 4 weeks. Blood was collected for measurements of serum levels of cholesterol, HDL-C, and LDL-C.

For studies in hepatocyte-specific *Abca1g1*-deficient mice, 10- to 14-week-old *Abca1 ^fl/fl^Abcg1 ^fl/fl^* mice received a single intravenous dose of 1 × 10^11^ vg of AAV-NGM282 in combination with 3 × 10^11^ vg of AAV-thyroxine-binding globulin (TBG)-Cre recombinase or a control virus encoding green fluorescent protein through the tail vein. *Abca1 ^fl/fl^Abcg1 ^fl/fl^* mice served as WT controls. AAV-TBG-Cre drives Cre recombinase expression under TBG promoter, which allows hepatocyte-specific expression. Four weeks after AAV administration, serum levels of cholesterol, HDL-C, and LDL-C were measured.

For studies in *Fgfr4*-deficient mice, *Fgfr4*^−/−^ mice were backcrossed to C57BL/6 mice for at least 10 generations, and age- and gender-matched WT C57BL6 mice were used as *Fgfr4*^+/+^ controls. AAV-FGF19 (3 × 10^11^ vg) or a control virus encoding green fluorescent protein was injected intravenously in a volume of 200 μl to 6- to 8-week-old female *Fgfr4*^+/+^ or *Fgfr4*^−/−^ mice. Mice were euthanized 12 months post AAV administration for cholesterol, HDL-C, LDL-C, and body weight measurements. For liver tumor assessment, the maximum diameter of liver tumor nodules in each mouse was measured with a caliper and total numbers of tumor nodules per liver were recorded. Livers were weighed and collected for histological examination.

For studies in *Apoe*-deficient mice, 12- to 14-week-old *Apoe*^−/−^ mice received a single 200 μl intravenous injection of 1 × 10^11^ vg AAV-NGM282 or a control virus encoding green fluorescent protein via tail vein. Mice were placed on a high-fat high-cholesterol Western diet (Teklad TD88137) immediately following AAV injection, and this diet was continued ad libitum throughout the study. Mice were euthanized 18 weeks after AAV administration for en face and histology analysis.

For studies in *Ldlr*-deficient mice, 10-week-old *Ldlr*^−/−^ mice received a single 200 μl intravenous injection of 1 × 10^11^ vg AAV-NGM282 or a control virus encoding green fluorescent protein via tail vein. Mice were placed on a standard chow diet ad libitum throughout the study. Serum cholesterol, HDL-C, and LDL-C were analyzed 4 weeks after AAV administration.

### DNA constructs

Human FGF19 cDNA (NM005117) was subcloned into pAAV-EF1α vector using *Spe*I and *Not*I sites with primers 5′-CCGACTAGTCACCATGCGGAGCGGGTGTGTGG-3′ (sense) and 5′-ATAAGAATGCGGCCGCTTACTTCTCAAAGCTGGGAC­TCCTC-3′ (antisense). Construct for NGM282 was generated by Quick-Change site-directed mutagenesis kit (Agilent Technologies). cDNAs for TBG promoter and Cre recombinase (AF298789) were chemically synthesized (DNA2.1) and subcloned into promoterless pAAV vector. cDNAs for caMEK1 (with EE mutations in the activation loop) and caSTAT3 were chemically synthesized (DNA2.1) and subcloned into pAAV-EF1α vector.

### AAV production

AAV293 cells (Agilent Technologies) were cultured in DMEM (Mediatech) supplemented with 10% FBS and 1× antibiotic antimycotic solution (Mediatech). Cells were cultured in a humidified incubator with 5% CO_2_ and 95% air at 37°C, confirmed to be mycoplasma free, and authenticated by short tandem repeat DNA profiling. The cells were transfected with three plasmids [AAV transgene, pHelper (Agilent Technologies), and AAV2/9] for viral production. Viral particles were purified using a discontinuous iodixanol (Sigma) gradient and resuspended in PBS with 10% glycerol and stored at −80°C. Viral titer or vector genome number was determined by quantitative PCR using custom TaqMan assays specific for woodchuck hepatitis virus posttranscriptional regulatory element (WPRE) sequences. Standard curves for WPRE were obtained from serial dilutions over a 6 log range of the corresponding plasmids. AAV-mediated gene delivery provides a means to achieve long-lasting transgene expression without the inflammatory responses that are commonly associated with other viral vectors. When introduced into adult mice, sustained expression of up to 1 year has been observed. The major site of transgene expression is the hepatocytes.

### Transcriptome profiling and pathway analysis

RNA was isolated from the livers of *db/db* mice 2 weeks after administration of AAV-FGF19 or a control virus and treated with DNase I (Thermo Fisher Scientific). RNA integrity and purity were confirmed by Bioanalyzer (Agilent Technologies) with RIN numbers >8.0. The raw expression data from Affymetrix mouse gene 1.0 ST whole-transcript arrays (Thermo Fisher) were normalized using the robust multi-array average method. The metadata and matrix tables have been deposited to the Gene Expression Omnibus (GEO) repository (accession number GSE117855). Ingenuity Pathway Analysis (IPA) (Qiagen), including canonical pathways, upstream analysis, diseases, and functions, was conducted on genes differentially represented in FGF19-treated versus control livers. The top canonical pathways were ranked by −Log (*P* value) with a threshold *P* value of 0.05. The highest ranking categories were sorted in a decreasing order of significance.

### Databases and bioinformatics

The Gene Tissue Expression (GTEx) project collected tissue samples from 554 human donors and carried out RNA-seq and other genomic profiling on these tissue samples. All GTEx data­sets used in the analyses described here are available through the GTEx portal (http://gtexportal.org). The donor samples with gene expression data are summarized in supplemental Table S3. Cardiovascular disease-related datasets (supplemental Table S4) were extracted from OmicSoft DiseaseLand database (Qiagen), which contains datasets retrieved from a variety of public projects including GEO, Sequence Read Archive, ArrayExpress, and the Database of Genotypes and Phenotypes. Bioinformatics analysis, including gene expression correlation and disease versus normal comparison related to cardiovascular diseases, was conducted using ArrayStudio software version 10.0 from OmicSoft (Qiagen).

### Gene expression by quantitative reverse transcription PCR

Mouse livers or ileum were snap-frozen in liquid nitrogen upon euthanization of animals. Total RNA was extracted using RNeasy Mini kit (Qiagen) and treated with DNase I (Thermo Fisher Scientific). Quantitative PCR with reverse transcription (qPCR) assays were performed using QuantiTect multiplex qRT-PCR master mix (Qiagen) and premade TaqMan gene expression assays (Life Technologies). Samples were loaded into an optical 384-well plate and qPCR was performed in duplicates on QuantStudio 7 Flex real-time PCR system (Applied Biosystems). After an initial hold at 50°C for 30 min to allow reverse transcription to complete, HotStart Taq DNA polymerase was activated at 95°C for 15 min. Forty cycles of a three-step PCR (94°C for 45 s, 56°C for 45 s, and 76°C for 45 s) were applied, and the fluorescence intensity was measured at each change of temperature to monitor amplification. Target gene expression was determined using the comparative threshold cycle (ΔΔCt) method and normalized to the expression of the housekeeping gene, GAPDH.

### Blood parameters

Blood was collected from the tail vein in unanesthetized animals using Microvette serum gel tubes (Sarstedt) for measurements of total cholesterol, HDL-C, LDL-C, aspartate aminotransferase (AST), and FGF19 concentrations. Serum samples were prepared by centrifugation at 4°C for 10 min at 2,000 *g* after clotting at room temperature for 30 min. Concentrations of total cholesterol, HDL-C, and LDL-C were measured by enzymatic methods using COBAS INTEGRA enzyme kit and an automated analyzer (COBAS INTEGRA 400 Plus clinical analyzer; Roche Diagnostics). In these enzymatic methods, esterified cholesterol is converted to free cholesterol by cholesterol esterase. The resulting cholesterol is then oxidized by cholesterol oxidase to produce Δ4-cholestenone and hydrogen peroxide. The hydrogen peroxide then reacts with 4-aminoantipyrine in the presence of peroxidase to produce a colored product that is measured at 583 nm. Direct determination of HDL-C is achieved through the combination of polyethylene glycol-modified enzymes, α-cyclodextrin sulfate, and magnesium chloride, which provides selectivity for HDL-C. The results of this method correlate with those obtained by precipitation-based or ultracentrifugation methods (19). Direct determination of LDL-C is achieved through the selective micellar solubilization of LDL-C by a nonionic detergent and the interaction of a sugar compound and lipoproteins. The combination of a sugar compound with detergent enables the selective determination of LDL-C in serum (Roche Diagnostics). These reagents are compatible for quantification of total cholesterol, HDL-C, and LDL-C levels in samples of human, mouse, or rat origin.

For fractionation of serum lipoproteins, pooled mouse serum samples (50 μl) were injected on two Superose 6 HR 10/30 columns connected in series on an AKTA Explorer fast protein LC system (GE Healthcare). Lipoproteins were eluted at a constant 0.3 ml min^−1^ flow rate with PBS (pH 7.4) containing 0.02% EDTA. Individual fractions were collected for total cholesterol measurements.

FGF19 and NGM282 concentrations were determined by FGF19 ELISA (Biovendor; RD191107200R). All assays were performed according to the manufacturers’ instructions. Blood concentrations of ad libitum-fed glucose were measured in conscious animals from a hand-held glucometer (Accu-check; Roche Diagnostics) using tail vein blood.

### Liver triglyceride and cholesterol content

Frozen liver samples (∼100 mg each) were homogenized in chloroform/methanol (2:1, v/v) using the Folch method. Aliquots of extracts were washed with isotonic saline, and the chloroform layers were dried under nitrogen gas. The lipids were reconstituted in isopropanol, and concentrations of total triglyceride and cholesterol were measured using Infinity liquid stable reagents (Thermo Fisher). Values were expressed as milligrams of triglyceride or cholesterol per gram wet weight of liver (mg g^−1^).

To determine levels of free cholesterol and hydroxycholesterol in the liver, frozen liver samples in glass tubes combined with internal standard were extracted with chloroform/methanol (2:1, v/v) (Creative Dynamics). Phospholipids were removed by SPE columns (Waters). The chromatographic system was a Michrom Paradigm HPLC equipped with a C18AQ analytical column (2.0 × 150 mm, 4 μm), an autosampler, a helium degassing system, and a column oven. Column eluent was introduced to a LTQ-Orbitrap Velos mass spectrometer (Thermo Fisher) with a heated electrospray ionization source. Retention times for each sterol were compared with reference standards (Steroloids Inc.).

### Histology

Mouse livers or hearts were fixed in 10% neutral-buffered formalin and embedded in paraffin. Five micron sections were deparaffinized in xylenes (5 min) and rehydrated sequentially in graded ethanol (100, 95, 80, 70, and 50%; 2 min each) and PBS (2 min). Stains with H&E, hematoxylin-phloxine-saffron (HPS), Masson’s trichrome, or von Kossa were performed using standard methods. For osmium tetroxide staining of lipids in the liver, frozen livers were embedded in optimal cutting temperature compound and directly processed for staining. For immunohistochemistry, specimens were subjected to antigen retrieval in a citrate-based antigen unmasking solution (Vector Laboratories; #H-3300) and incubated for 30 min with 3% H_2_O_2_ at room temperature to block endogenous peroxidase activity. Sections were blocked in PBST (PBS + 0.1% Tween-20) containing 10% goat serum and stained with primary antibodies against glutamine synthetase (Abcam), CD68, or smooth muscle actin diluted in blocking solution at 4°C overnight. Specimens were then washed three times for 5 min each in PBST and incubated with biotinylated secondary antibodies in blocking solution for 1 h at room temperature. ImmPRESS-alkaline phosphatase reagent (Vector Laboratories) and ImmPACT Vector Red substrate or ABC reagent with 3,3-diaminobenzidine substrate were used for detection. Digital imaging microscopy was performed using a Leica DM4000 microscope equipped with a DFC500 camera and a high-precision motorized scanning platform (Leica). Images for the entire liver section were acquired by Turboscan and real-time imaging stitching at camera frame rates using the Surveyor program. For morphometric analysis of tumor area, glutamine synthetase-positive tumor areas were quantified using the measure/count/area tool from ImagePro software.

### En face studies in atherosclerotic *Apoe*^−/−^ mice

*Apoe*^−/−^ mice on a high-fat high-cholesterol Western diet, an established model for atherosclerosis with features of aortic plaque and lesion formation, were used for the evaluation of the effects of NGM282 on atherosclerosis development. At 18 weeks on Western diet, mice were euthanized and atherosclerosis development in aortas and aortic roots was measured. En face analysis was conducted at Wake Forest School of Medicine Metabolic Core (Winston-Salem, NC). Briefly, the aortas were cleaned of any adventitial and fat tissue, cut open longitudinally, and pinned flat on a black wax surface. Aorta images were captured through a stereomicroscope (Leica S8 APO) with a digital camera (Leica MC-120). Lesion area was quantified using ImageJ software according to published protocols and expressed as percent stained area relative to total aortic area. All quantifications were carried out by an observer blinded to the sample identity. No animals were excluded from atherosclerotic lesion analysis. Aortic contents of total cholesterol, free cholesterol, and cholesterol ester were quantified at Wake Forest School of Medicine Metabolic Core Facility.

### Aortic root sectioning and staining

Hearts from *Apoe*^−/−^ mice treated with NGM282 or control mice were isolated, fixed in formalin, and embedded in paraffin. Mouse hearts were sectioned perpendicular to the axis of the aorta, and once the aortic root was identified by the appearance of aortic valve leaflets and smooth muscle cells, cross-sections (5 μm thick) were taken and mounted on AAS-coated slides. The sections were stained with HPS for necrotic area and Masson’s trichome for collagen content. The images were recorded using Aperio software.

### NGM282 clinical trial in healthy volunteers

A phase 1, first-in-human, randomized, placebo-controlled, double-blind clinical trial was performed to assess the safety, tolerability, and pharmacodynamics of NGM282 administered subcutaneously to healthy volunteers. The study was conducted in accordance with the Declaration of Helsinki and with Good Clinical Practice guidelines. The Human Research Ethics Committee approved the study protocol. Written informed consent was obtained from all subjects prior to participation. Treatment allocation and concealment were conducted by a computerized random-number generator and numbered containers with active and placebo syringes of identical appearance. The randomization list was centrally held by an independent contract research organization. All randomized subjects, the sponsor, the study center, and contract research organization personnel were blinded to treatment allocation until data were locked and analyzed.

After a screening period, participants were randomized to receive 3 mg NGM282 protein (n = 9) or placebo (n = 17) subcutaneous treatment once daily for 7 days. The sample size chosen was based on precedents set by other first-in-human studies of a similar nature for exploratory safety evaluation and exposure assessment. Study visits occurred during the screening period, on day 1, day 3, and the last day of treatment (day 7). Blood sampling was performed in the fasting state at each visit for lipid measurements.

Study participants were healthy volunteers of 18–65 years of age, with BMI of 25–35 kg/m^2^. No clinically significant findings were noted from medical history, physical examination, twelve-lead ECG, clinical laboratory, and vital signs at screening. Patients were excluded if they demonstrated a history or the clinical manifestation of any significant metabolic, allergic, dermatological, hepatic, renal, hematological, pulmonary, cardiovascular, gastrointestinal, neurological, or psychiatric disorders. This trial is registered with clinicaltrials.gov, number NCT01776528.

### Statistical analysis

For studies in mice, experiments were conducted with cohorts of 4–18 mice per group (n values are detailed in the figure legends), with individual mouse data shown when indicated. All results are expressed as mean ± SEM. One-way ANOVA followed by Dunnett’s posttest was used to compare data from multiple groups (GraphPad Prism). Unpaired two-tailed Student’s *t*-test was used to compare two treatment groups (GraphPad Prism). A *P* value of 0.05 or less was considered statistically significant.

For the clinical trial in humans, we used SAS (version 9.4) for all analyses. The difference between NGM282 and placebo groups was analyzed using ANCOVA model with treatment group as the factor and baseline values of the outcome as cofactor. All statistical analyses were carried out using two-sided tests at the 5% level of significance. Least-squares means, difference in least-squares means, 95% CIs for the difference, and corresponding *P* values were presented.

### Data availability

The GTEx and GEO datasets (see supplemental Table S4 for a list of datasets with accession numbers) used in the study are available in public repositories (http://gtexportal.org for GTEx, http://www.ncbi.nlm.nih.gov/gds for Gene Expression Omnibus). Graphic artwork was created using Biorender software. All other data supporting the findings of this study are available within the article and its supplemental information files.

## RESULTS

### FGF19 and NGM282 selectively modulate hepatic LXR signaling without inducing hepatic steatosis in diabetic *db/db* mice

**Fig. 1.**
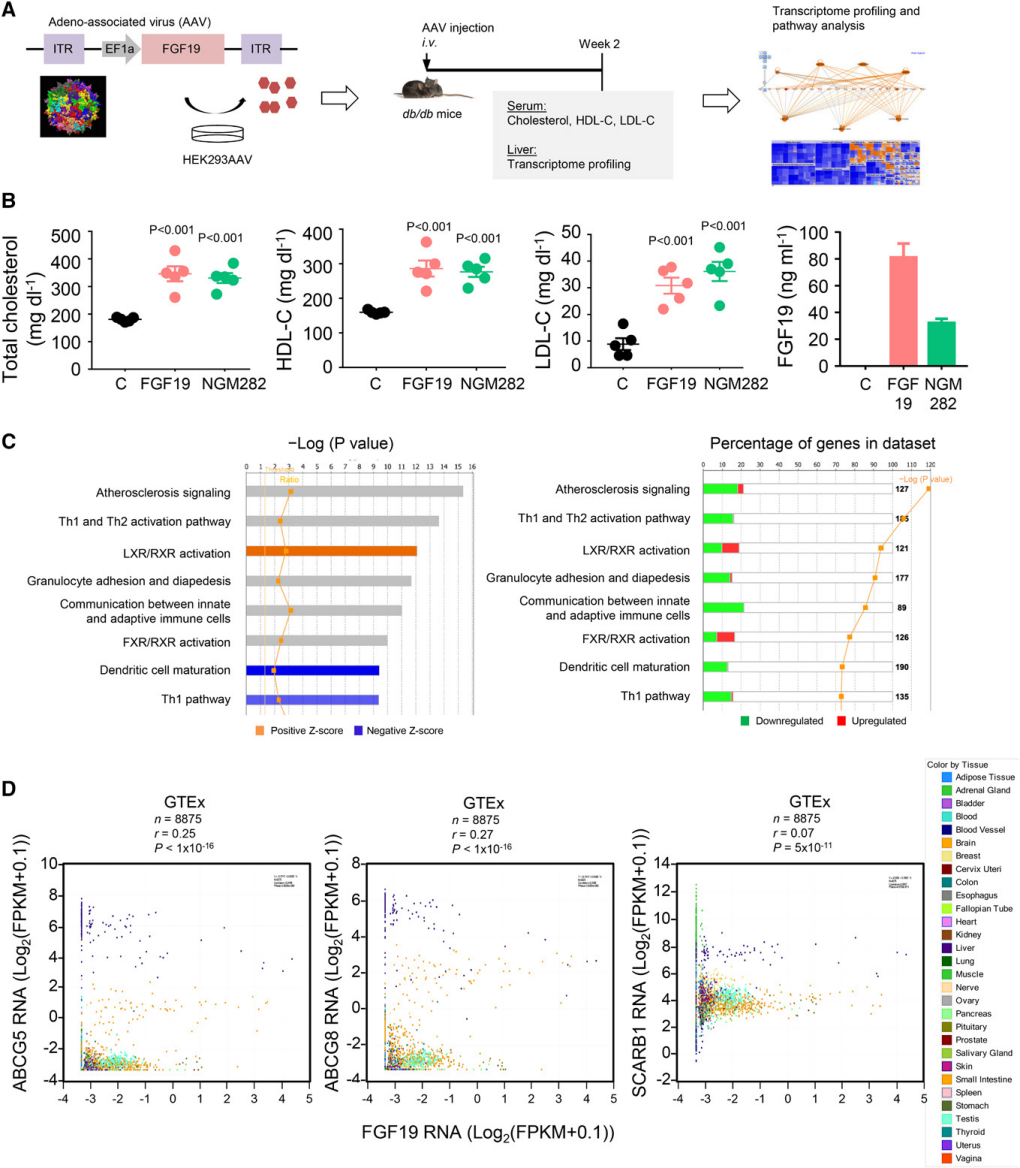
FGF19 and NGM282 increase serum cholesterol concentrations in *db/db* mice. A: The configuration of the AAV carrying the FGF19 transgene and study design. Genome-wide transcriptome profiling was performed on the livers from *db/db* mice 2 weeks after intravenous (*i.v*.) injection of AAV-FGF19 or a control virus carrying green fluorescent protein. ITR, inverted terminal repeat; EF1α, elongation factor 1α promoter. B: Serum concentrations of total cholesterol, HDL-C, LDL-C, and FGF19 in *db/db* mice 2 weeks after intravenous injection of AAV-FGF19 (n = 5 mice), AAV-NGM282 (n = 5 mice), or a control (C) virus carrying green fluorescent protein (n = 5 mice). Data are mean ± SEM, numbers on the graphs are *P* values versus the control group by one-way ANOVA with Dunnett’s posttest. C: Transcriptome profiling and IPA of livers from *db/db* mice 2 weeks after AAV-FGF19 injection. Top enriched canonical pathways are ranked by –Log (*P* value) with threshold *P* value of 0.05. The highest ranking categories are displayed along the x axis in decreasing order of significance. Orange bars, pathways with Z-score >0 (upregulated pathways); blue bars, pathways with Z-score <0 (downregulated pathways); gray bars, no activity pattern available. The percentage of genes in the datasets was summarized in the right panel. D: Correlation of FGF19 and LXR target genes (*ABCG5*, *ABCG8*, and *SCARB1*) in the GTEx database (n = 8,875 biologically independent human tissue samples). Expression values were Log2-transformed [Log_2_(FPKM + 0.1)], where FPKM is the number of fragments per kilobase of transcript per million mapped reads.

To explore the in vivo role of FGF19 in cholesterol homeostasis in the context of diabetes, a global health concern that is frequently associated with dyslipidemia, we introduced and expressed FGF19 transgenes in diabetic *db/db* mice by intravenous AAV delivery methods (**Fig. 1A**). The *db/db* model allows simultaneous assessment of blood cholesterol and glucose levels (data not shown). Two weeks after AAV injection, mice expressing either WT FGF19 or NGM282 showed significant elevations in serum concentrations of total cholesterol, HDL-C, and LDL-C compared with *db/db* mice injected with a control virus expressing green fluorescent protein (Fig. 1B). Circulating FGF19 and NGM282 concentrations were 81.1 ± 10.3 ng ml^−1^ and 32.2 ± 3.0 ng ml^−1^, respectively.

To understand the molecular mechanism by which serum levels of cholesterol increase in response to FGF19, we performed genome-wide transcriptome profiling on the livers from mice expressing FGF19 transgene. Among genes differentially regulated by FGF19, IPA revealed several enriched pathways [the “LXR/retinoid X receptor (RXR) activation” pathway in particular, with a Z-score of 1.932 (*P* < 1 × 10^−12^)] (Fig. 1C; supplemental Tables S1, S2; supplemental Fig. S1). Further analysis of the GTEx database revealed that the expression of FGF19 correlated with RNA levels of LXR target genes [ABCG5, ABCG8, and scavenger receptor B1 (SCARB1)] in tissue samples (n = 8,875) from human donors (Fig. 1D, supplemental Table S3).

**Fig. 2.**
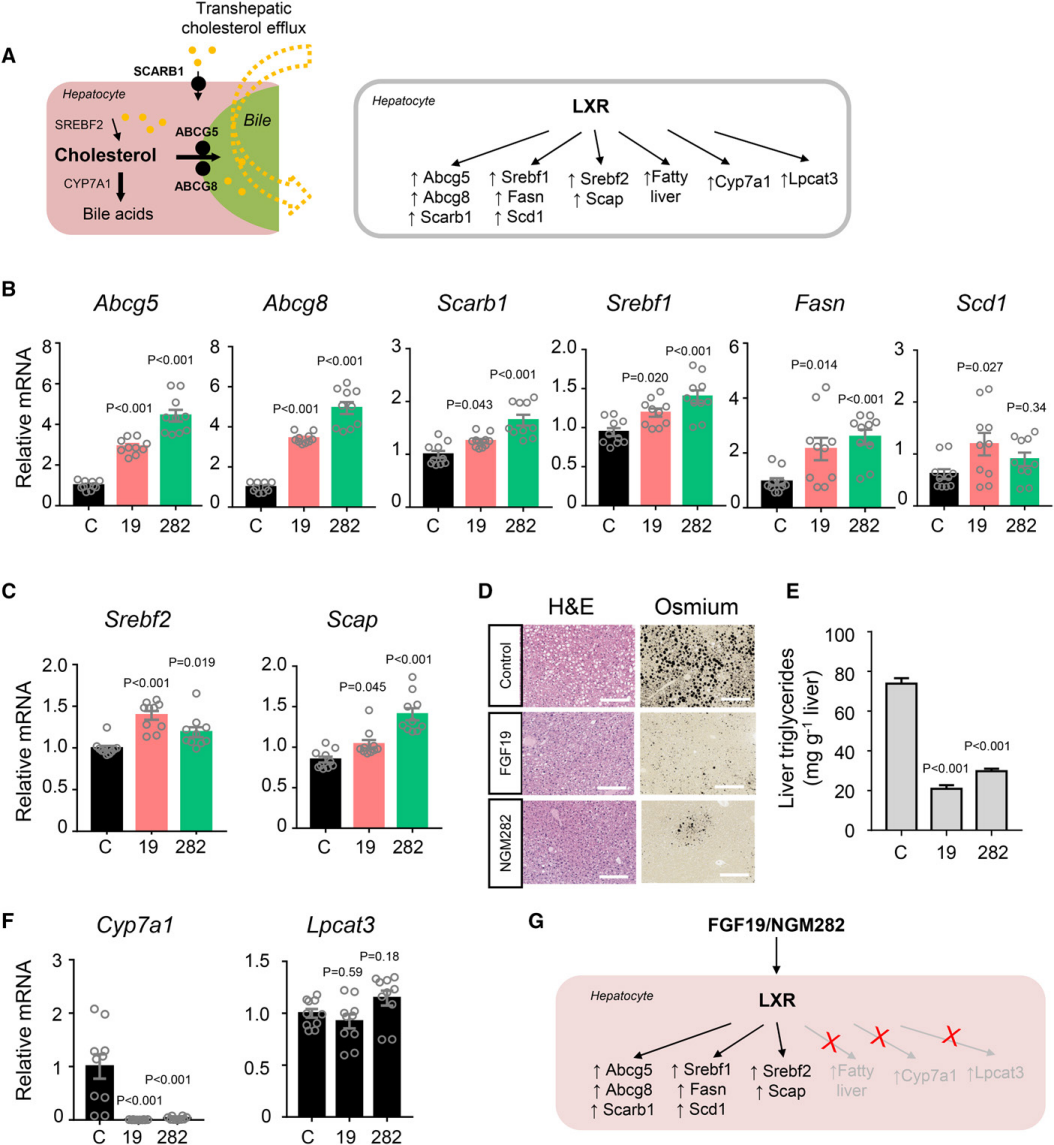
FGF19 and NGM282 selectively activate hepatic LXR signaling without causing steatosis. A: Transhepatic cholesterol efflux showing cholesterol uptake into the liver by SCARB1 and efflux into bile by ABCG5/8 (left panel). In addition to control genes responsible for transhepatic cholesterol efflux, LXR also regulates genes important for cholesterol, fatty acids, and bile acid metabolism (right panel). B, C: qPCR analysis of mRNA levels of LXR target genes that were upregulated by FGF19 and NGM282. Livers were harvested from *db/db* mice 2 weeks after intravenous injection of AAV-FGF19 (19), AAV- NGM282 (282), or a control virus (C) (n = 10 biologically independent samples per group). D: Representative osmium tetroxide staining of livers. Lipid droplets are shown as black vacuoles by osmium tetroxide method (n = 5 mice per group). H&E staining was also included (scale bars, 100 μm). E: Hepatic triglyceride content by Folch method (n = 5 mice per group). F: qPCR analysis of LXR target genes that were not upregulated by FGF19 and NGM282 (n = 10 biologically independent samples per group). G: Cartoon showing selective activation of LXR targets by FGF19 and NGM282. Data are mean ± SEM, numbers on the graphs are *P* values versus the control group by one-way ANOVA with Dunnett’s posttest.

The LXRs are nuclear receptors that activate genes involved in reverse cholesterol transport from peripheral tissues to the liver (20). Once taken into the liver via SCARB1 (also known as SR-BI) (21), which contains a LXR/RXR response element in its promoter (22), cholesterol can be directly excreted from the liver into the bile via the ABC half transporters, ABCG5 and ABCG8 (23) (**Fig. 2A**). Given the importance of LXRs as transcriptional regulators of cellular and systemic cholesterol homeostasis, we used a qPCR assay to compare the hepatic mRNA levels of key LXR target genes in *db/db* mice treated with AAV-FGF19, AAV-NGM282, or a control virus. Notably, the genes most robustly induced following FGF19 and NGM282 treatment were *Abcg5* and *Abcg8* (Fig. 2B); mRNA levels of *Scarb1* were also increased in mice expressing either the FGF19 or NGM282 transgene. Based on transcriptional profiling, FGF19 and NGM282 promote transhepatic efflux of cholesterol by upregulating the LXR target genes, *Abcg5*, *Abcg8*, and *Scarb1*.

LXRs also facilitate cholesterol esterification by promoting the synthesis of fatty acids and triglycerides via upregulation of SREBP-1c, the master regulator of fatty acid synthesis (24, 25). As shown in Fig. 2B, treatment of *db/db* mice with FGF19 or NGM282 increased hepatic expression of *Srebf1*, *Fasn*, and *Scd1*. Additionally, FGF19 and NGM282 expression also increased the mRNA levels of SREBP-2, the transcription factor that regulates cholesterol synthesis and transport (Fig. 2C). There was also marked induction of SREBP cleavage-activating protein (SCAP), which accelerates SREBP processing through proteolytic cleavage to the smaller transcriptionally active form (26). Consistent with the notion that LXRs repress lipopolysaccharide induction of inflammatory mediators (27), we observed marked reduction in pro-inflammatory cytokine expression (such as *Il1a*, *Il1b*, *Tnf*, *Ccl2*, and *Ccl5*) and pattern recognition receptor signaling in FGF19-treated livers (supplemental Fig. S1).

LXR activation by synthetic agonists has traditionally been associated with profound hepatic steatosis (28, 29). However, hepatic steatosis was ameliorated, rather than increased, following FGF19 or NGM282 treatment of *db/db* mice, despite increases in *Srebf1*, *Fasn*, and *Scd1* mRNA levels (Fig. 2D, E). Thus, FGF19 and NGM282 appear to selectively modulate LXR signaling, upregulating canonical LXR target genes (*Abcg5*, *Abcg8*, *Scarb1*, *Srebf1*, *Fasn*, *Scd1*, *Srebf2*, and *Scap*) without inducing hepatic steatosis.

LXR also controls cholesterol catabolism by upregulating CYP7A1, a key enzyme in the classic pathway of bile acid synthesis (30, 31), and increasing the conversion of cholesterol into bile acids. Consistent with the evidence of selective LXR modulation presented in this report, as well as data from previous studies (15, 18), *Cyp7a1* mRNA levels were decreased, rather than induced, by FGF19 and NGM282 (Fig. 2F). Moreover, no significant differences in the hepatic mRNA levels of the LXR target gene, lysophosphatidylcholine acyltransferase 3 (*Lpcat3*, also known as *Mboat5*), were observed in *db/db* mice treated with FGF19 or NGM282, compared with control animals (Fig. 2F, G). Intrahepatic content of cholesterols and oxysterols such as 27-hydroxycholesterol was elevated in FGF19- or NGM282-treated animals (supplemental Fig. S2), potentially providing a mechanism for LXR activation, as cholesterol metabolites form natural ligands of LXR (32). The selective activation of LXR signaling by FGF19 and NGM282 appears to be specific to the liver, as the expression of LXR target genes (*Abcg5*, *Srebf1*, *Fasn*, and *Srebf2*) was unchanged in the ileum of these mice (supplemental Fig. S3).

Collectively, these data show that both FGF19 and NGM282 selectively activate hepatic LXR signaling to regulate transhepatic cholesterol efflux, a key step in reverse cholesterol transport, without causing steatosis.

### FGF19 and NGM282 promote biogenesis of nascent HDL via hepatocellular ABCA1

FGF19- and NGM282-associated induction of Scarb1, a key player in reverse cholesterol transport responsible for hepatic uptake of HDL (33), would predict increased clearance rates and the consequent lowering of circulating HDL-C levels. Paradoxically, as shown in Fig. 1B, we observed a rise in serum HDL-C levels in mice expressing FGF19 or NGM282, indicating that other mechanisms might be in play.

**Fig. 3.**
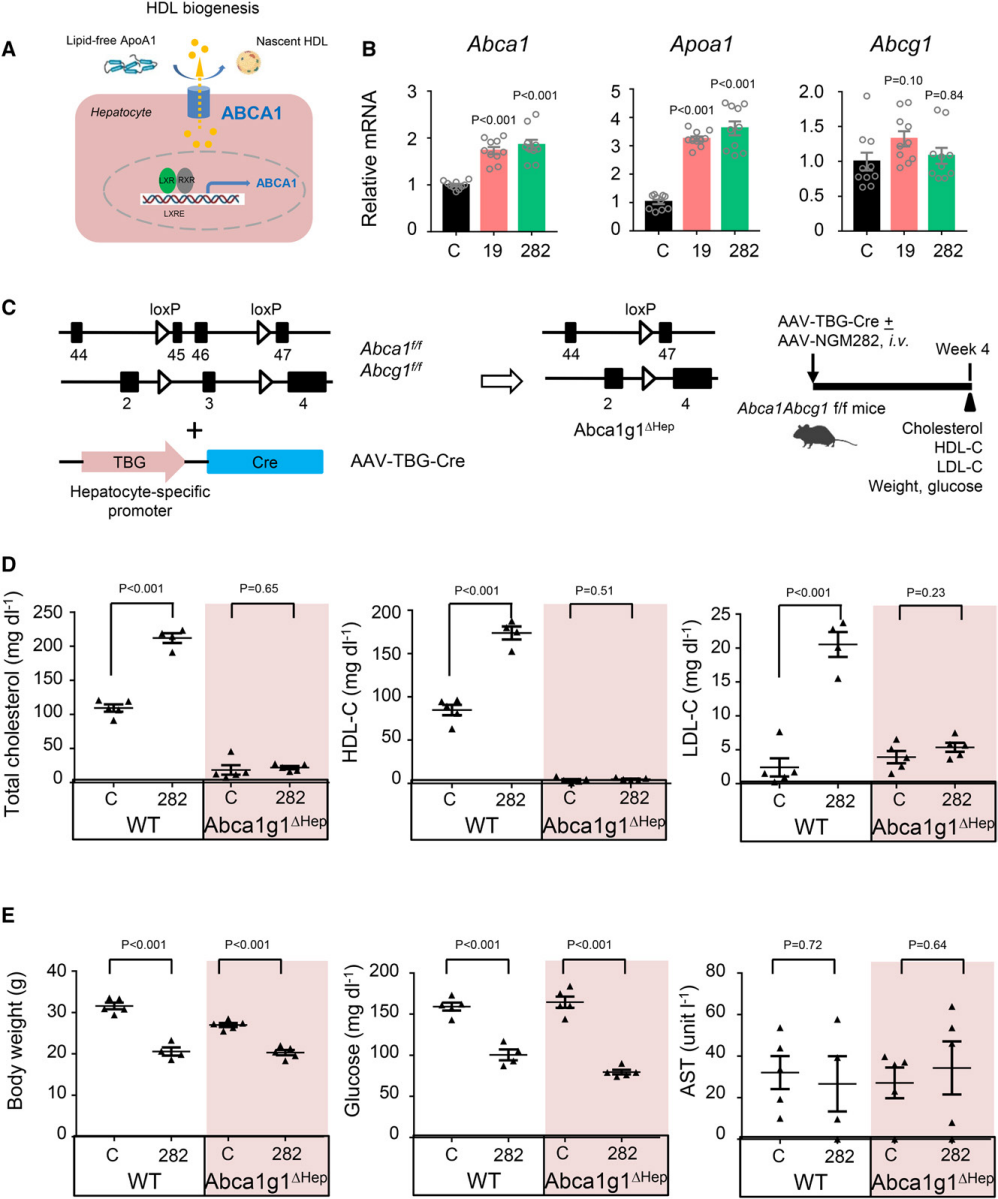
FGF19 and NGM282 induce *Abca1* to promote HDL biogenesis. A: Nascent HDL formation mediated by hepatic ABCA1, which is controlled by LXR signaling. B: Upregulation of *Abca1* and *Apoa1* by FGF19 and NGM282 by qPCR analysis. Livers were harvested from *db/db* mice 2 weeks after intravenous injection of AAV-FGF19 (19), AAV-NGM282 (282), or a control virus (C) (n = 10 biologically independent samples per group). C: Generation of *Abca1g1^ΔHep^* mice using Cre recombinase driven by the TBG promoter. D: Hepatocellular deficiency of ABCA1 and ABCG1 abolishes NGM282-associated increases in total cholesterol, HDL-C, and LDL-C. Serum concentrations of total cholesterol, HDL-C, LDL-C were measured 4 weeks after intravenous injection of AAV-NGM282, or a control (C) virus, with or without AAV-TBG-Cre. WT mice treated with control virus (n = 5 mice); WT mice treated with NGM282 (n = 4 mice); *Abca1g1^ΔHep^* mice treated with control virus (n = 5 mice); *Abca1g1^ΔHep^* mice treated with NGM282 (n = 5 mice). E: Hepatocellular deficiency of ABCA1 and ABCG1 has no impact on NGM282-associated effects on body weight, glucose, or AST levels. Data are mean ± SEM; numbers on the graphs are *P* values versus the control group by one-way ANOVA with Dunnett’s posttest for multi-group comparison or unpaired two-tailed *t*-test.

There is long-standing evidence that the liver is the major site of HDL production, contributing >80% of circulating HDL levels (34). Efflux of hepatic cholesterol to the serum compartment by ABCA1 for the production of nascent HDL is an important pathway for maintenance of plasma cholesterol levels. During HDL biogenesis, hepatocytes efflux cholesterol to lipid-free ApoA1 to form nascent HDL particles (**Fig. 3A**). qPCR analysis of livers harvested from *db/db* mice injected with AAV-FGF19 or AAV-NGM282 revealed marked elevation in mRNA levels of *Abca1* and *Apoa1*, and a minor increase in *Abcg1* (Fig. 3B).

To examine the role of ABCA1 and ABCG1 in the HDL increase associated with FGF19 and NGM282, we injected *Abca1^fl/fl^Abcg1^fl/fl^* mice with AAV-TBG-Cre to drive hepatocyte-specific Cre recombinase expression from the TBG promoter and establish mice deficient for ABCA1 and ABCG1 in hepatocytes (referred to as *Abca1g1^ΔHep^* mice in Fig. 3C–E). Previous studies have shown that ABCG1 is predominantly expressed in nonparenchymal cells (i.e., Kupffer cells and endothelial cells) in the liver, and hepatocellular ABCG1 does not play a significant role in nascent HDL particle formation (35). In contrast, the deletion of ABCA1 in hepatocytes resulted in a pronounced reduction in serum HDL-C concentrations, consistent with the major role of ABCA1 in HDL biogenesis. Treatment with NGM282 increased the levels of HDL-C and total cholesterol in WT mice, but not in *Abca1g1^ΔHep^* mice (Fig. 3D). Interestingly, while ablation of hepatocellular ABCA1 did not affect serum LDL-C levels in mice treated with a control virus, this deletion abolished NGM282-associated elevation in LDL-C at 4 weeks after AAV-NGM282 injection (Fig. 3D). In contrast, deletion of ABCA1 in hepatocytes had no effect on the weight- and glucose-lowering activity of FGF19 and NGM282 (Fig. 3E), consistent with the notion that these metabolic effects are controlled by the FGFR1c-βKlotho receptor complex (14). Moreover, NGM282 reduced intrahepatic triglycerides in both WT and *Abca1g1^ΔHep^* mice without increasing circulating triglyceride levels (supplemental Fig. S4). No significant changes in liver enzyme AST were observed in these mice (Fig. 3E). Circulating NGM282 concentrations were 64.4 ± 16.8 ng ml^−1^ in these mice.

Taken together, these findings suggest that the induced hepatic expression of *Abca1* and *Apoa1*, which encode key proteins that promote HDL biogenesis, represents the major mechanism responsible for the elevated HDL-C levels observed in FGF19- or NGM282-treated mice.

### Effect of FGF19 on cholesterol is dependent on FGFR4 and mimicked by constitutive activation of ERK, but not STAT3, signaling

We further investigated whether the FGF19-mediated changes in blood cholesterol are dependent on FGFR4, which, together with the transmembrane protein βKlotho, form the primary receptor complex for FGF19 (8).

**Fig. 4.**
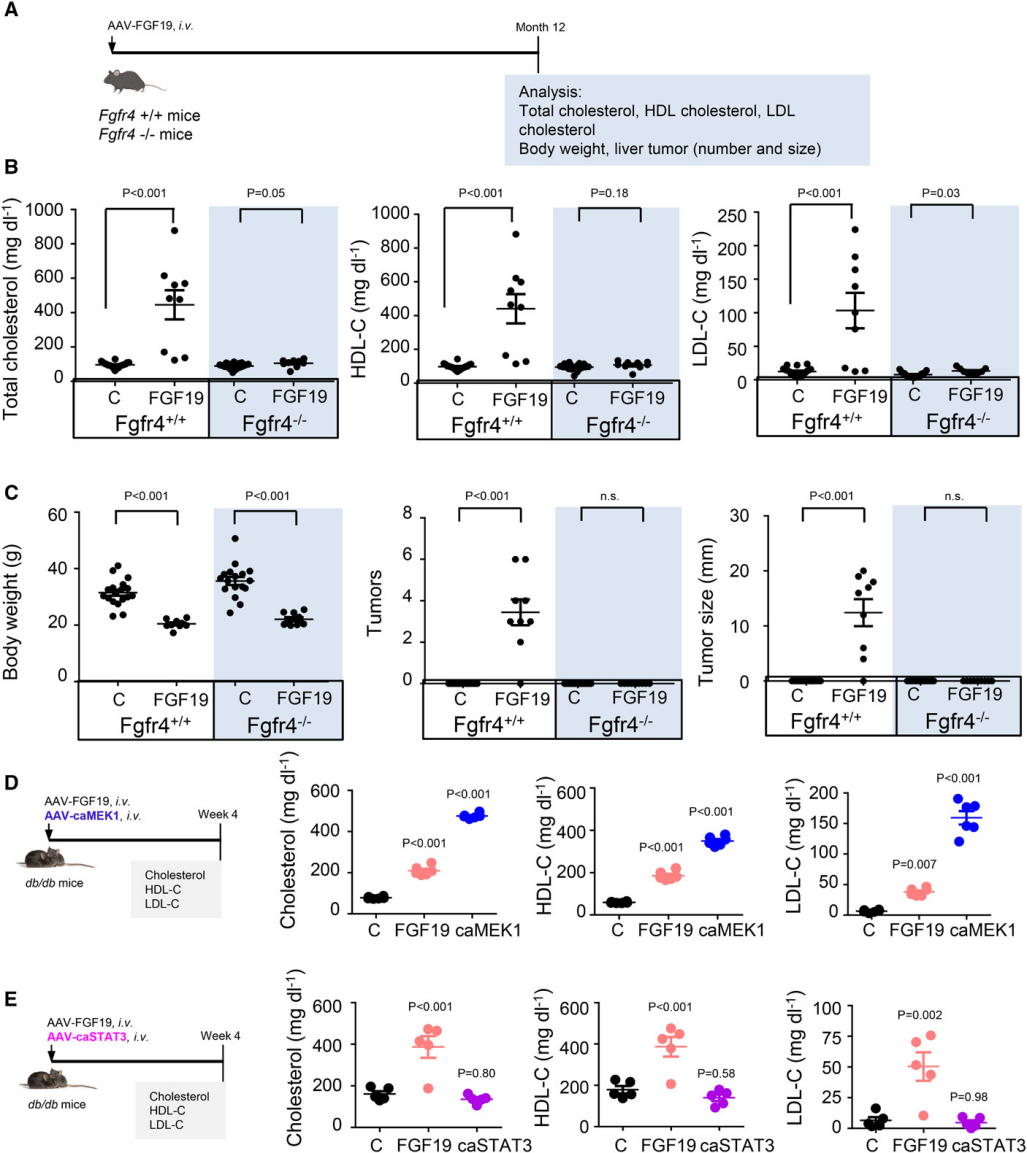
Effect of FGF19 on cholesterol is dependent on FGFR4 and mediated by ERK, but not STAT3, signaling. A: *Fgfr4*^+/+^ or *Fgfr4*^−/−^ mice were intravenously injected with AAV-FGF19 or a control (C) virus. Mice were euthanized 12 months later for analysis of serum cholesterol, body weight, and liver tumors. *Fgfr4*^+/+^ mice injected with control virus (n = 18 mice); *Fgfr4*^+/+^ mice injected with AAV-FGF19 (n = 9 mice); *Fgfr4*^−/−^ mice injected with control virus (n = 17 mice); *Fgfr4*^−/−^ mice injected with AAV-FGF19 (n = 9 mice). B: FGFR4 deficiency abolishes FGF19-associated increases in total cholesterol, HDL-C, and LDL-C. C: Deficiency of FGFR4 has no impact on FGF19-associated weight-lowering effects, but abolishes FGF19-associated hepatocarcinogenicity. The number of liver tumor nodules and maximum tumor diameter were recorded in each mouse. D: caMEK1 raises serum levels of total cholesterol, HDL-C, and LDL-C. The *db/db* mice received an intravenous injection of AAV-FGF19, AAV-caMEK1, or a control (C) virus and were analyzed 4 weeks later (n = 6 mice per group). E: caSTAT3 does not affect serum levels of total cholesterol, HDL-C, and LDL-C. The *db/db* mice received an intravenous injection of AAV-FGF19, AAV-caSTAT3, or a control (C) virus (n = 6 mice per group). Data are mean ± SEM; numbers on the graphs are *P* values versus the control group by unpaired two-tailed *t*-test or one-way ANOVA with Dunnett’s posttest for multi-group comparison. n.s., not significant.

To this end, AAV-FGF19 was administered to *Fgfr4*^+/+^ and *Fgfr4*^−/−^ mice (**Fig. 4A**). Consistent with the results obtained in *db/db* mice, FGF19 expression increased levels of total cholesterol, HDL-C, and LDL-C in *Fgfr4*^+/+^ mice. However, these effects were entirely abolished in *Fgfr4*-deficient mice (Fig. 4B). No difference was observed on the weight-lowering activity of FGF19 (Fig. 4C), consistent with the notion that FGF19 reduces body weight through the FGFR1c-βKlotho receptor complex, but not the FGFR4-βKlotho receptor complex (14). As expected, prolonged exposure to FGF19 induced liver tumor formation in *Fgfr4*^+/+^ mice. In contrast, genetic ablation of *Fgfr4* eliminated FGF19-induced tumor formation (Fig. 4C). Moreover, FGF19 expression increased liver weight as well as the liver-to-body weight ratio in *Fgfr4*^+/+^ mice, but not in *Fgfr4*^−/−^ mice (supplemental Fig. S5). Therefore, FGFR4 plays an essential role in mediating both the cholesterol-raising and tumorigenic effects of FGF19.

FGF19 has previously been shown to activate multiple intracellular signaling pathways, including the ERK-mediated repression of CYP7A1 expression and the STAT3 signaling to promote hepatocarcinogenesis (15, 18, 36). To examine the contribution of ERK and STAT3 signaling to FGF19-associated changes in cholesterol, we injected *db/db* mice with AAV carrying caMEK1 (37) or caSTAT3 (38). As shown in Fig. 4D, E, expression of caMEK1 significantly elevated serum cholesterol, HDL-C, and LDL-C levels, whereas expression of caSTAT3 had no effect on these parameters.

Furthermore, administration of a HMG-CoA reductase inhibitor (rosuvastatin) or a blocking antibody against PCSK9 (39), but not an inhibitor of Niemann-Pick C1-like 1 (NPC1L1) (ezetimibe), abolished FGF19-induced changes in total cholesterol, HDL-C, and LDL-C in *db/db* mice (supplemental Fig. S6). However, cholesterol elevation following NGM282 administration also occurred in *Ldlr*^−/−^ mice, suggesting that a functional LDLR signaling is not required (supplemental Fig. S7).

In summary, FGF19-induced cholesterol changes are dependent on FGFR4 and mimicked by the constitutive activation of ERK, but not STAT3, signaling.

### FGF19 analog NGM282 demonstrates anti-atherogenic activity in *Apoe*^−/−^ mice fed a Western diet

**Fig. 5.**
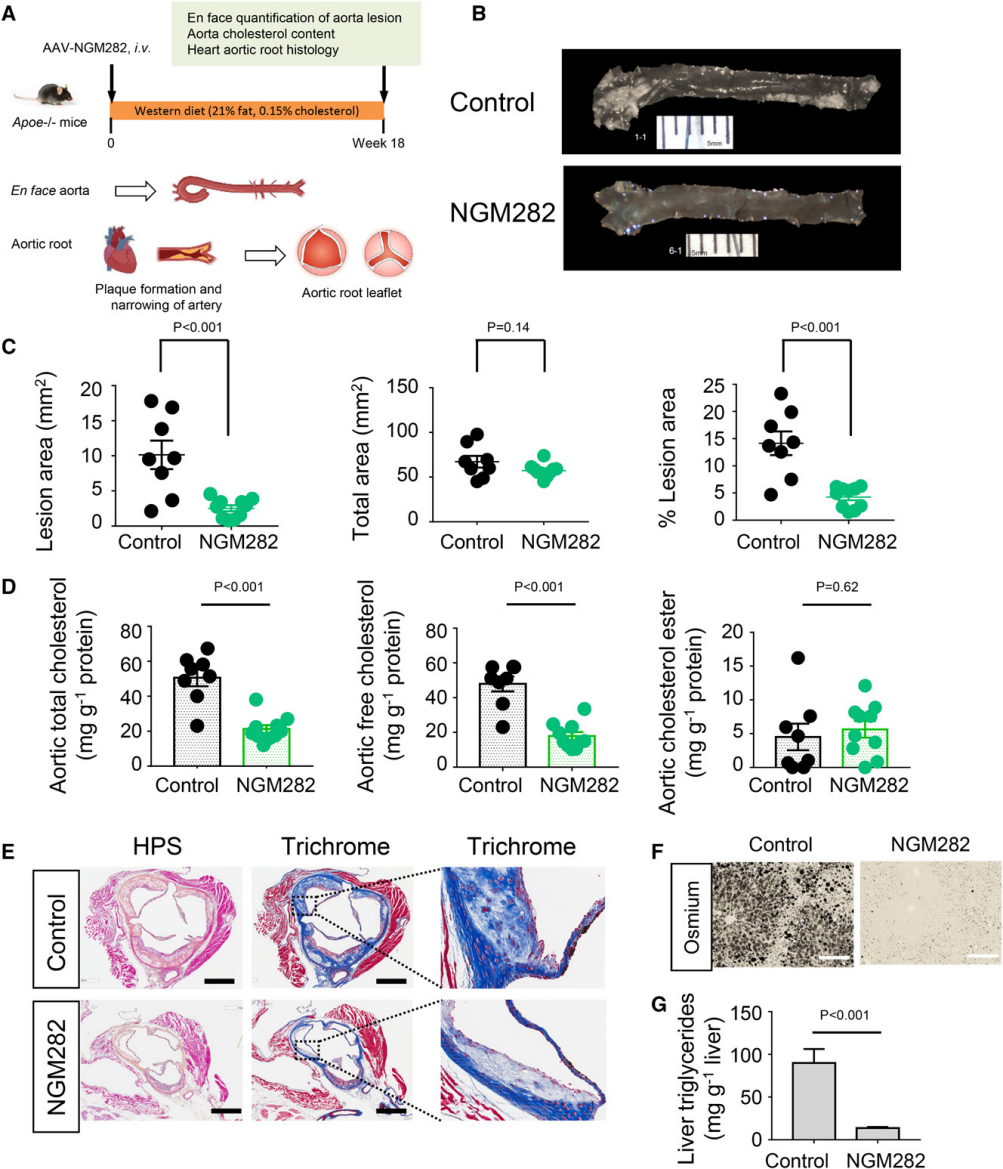
The FGF19 analog, NGM282, protects against atherosclerosis in *Apoe*^−/−^ mice fed a Western diet. A: *Apoe*^−/−^ mice received intravenous injection of AAV-NGM282 or a control virus, and were fed a high-fat (21% w/w fat), high-cholesterol (0.15% w/w cholesterol) Western diet for 18 weeks. Aortas were harvested for en face analysis, and hearts with aortic roots were stained as indicated. B: Representative images of aortas with atherosclerotic plaques for en face analysis. *Apoe*^−/−^ mice injected with control virus (n = 8 mice); *Apoe*^−/−^ mice injected with AAV-NGM282 (n = 10 mice). C: NGM282 reduces total atherosclerotic lesion area and percent atherosclerotic lesion area. *Apoe*^−/−^ mice injected with control virus (n = 8 mice); *Apoe*^−/−^ mice injected with AAV-NGM282 (n = 10 mice). D: NGM282 reduces aortic content of total cholesterol and free cholesterol. *Apoe*^−/−^ mice injected with control virus (n = 8 mice); *Apoe*^−/−^ mice injected with AAV-NGM282 (n = 10 mice). E: Representative images of atherosclerotic plaque formation in cross-sections of the aortic root area. Sections were stained with HPS or Masson’s trichrome as indicated. Fibrosis is visualized as blue color in trichrome stain. Magnifications of boxed regions are shown on the right (scale bars, 500 μm). F: Representative images of liver fat staining. Liver steatosis is evidenced as black vacuoles by osmium tetroxide stain (scale bars, 100 μm). G: Hepatic triglyceride content by Folch method. Data are mean ± SEM; numbers on the graphs are *P* values versus the control group by unpaired two-tailed *t*-test.

Given the role of LXR in regulating reverse cholesterol transport and atherosclerosis prevention (20), we evaluated the effect of NGM282 on atherosclerosis progression in *Apoe*^−/−^ mice fed a Western diet (21% w/w fat, 0.15% w/w cholesterol) for 18 weeks (**Fig. 5A**). As measured by en face analysis, treatment with NGM282 reduced atherosclerotic lesion area by 75% compared with treatment with a control virus (*P* < 0.001; Fig. 5B, C). In the aorta, only 4.3 ± 0.6% of the vessel surface was covered with atherosclerotic plaques in NGM282-treated *Apoe*^−/−^ mice, compared with 14.2 ± 2.2% in the control *Apoe*^−/−^ mice. Furthermore, the aortic content of total cholesterol and free cholesterol were reduced by 58% and 63%, respectively, in NGM282-treated *Apoe*^−/−^ mice (Fig. 5D). In contrast, no effects of NGM282 on the aortic content of cholesterol esters were observed in these mice. Circulating NGM282 concentrations were 51.0 ± 10.9 ng ml^−1^.

Finally, administration of NGM282 was associated with a decrease in both necrotic content and fibrosis, as revealed by the staining of aortic roots with HPS and Masson’s trichrome (Fig. 5E). No changes in aortic macrophage (anti-CD68), smooth muscle cell (anti-smooth muscle actin), or calcium (von Kossa) content were noted (supplemental Fig. S8A). Hepatic steatosis, as evidenced by osmium tetroxide staining and quantification of hepatic triglyceride content using the Folch method, was also reduced in NGM282-treated *Apoe*^−/−^ mice (Fig. 5F, G). As a high-cholesterol diet imposes a significant sterol load upon the mouse liver, these *Apoe*^−/−^ mice had elevated background LXR signaling. Consequently, we did not observe a further increase in circulating cholesterol or triglyceride-rich VLDLs by NGM282 (supplemental Fig. S8B, C).

To explore the translational relevance of our findings, we examined FGF19 RNA levels in patients with cardiovascular disease identified from the OmicSoft DiseaseLand database (a list of cardiovascular disease datasets is included in supplemental Table S4). Significant downregulation of FGF19 expression was observed in disease tissues when compared with normal tissues from multiple independent datasets (supplemental Table S5). These results corroborate previous reports that lower FGF19 concentrations were associated with the presence and severity of coronary artery diseases in humans (40).

Based on these results, FGF19 analog NGM282 protects *Apoe*^−/−^ mice from atherosclerosis following challenge with an atherogenic diet. The translational relevance of this finding to humans warrants further investigation.

### Effect of NGM282 on serum cholesterol in healthy human volunteers

**Fig. 6.**
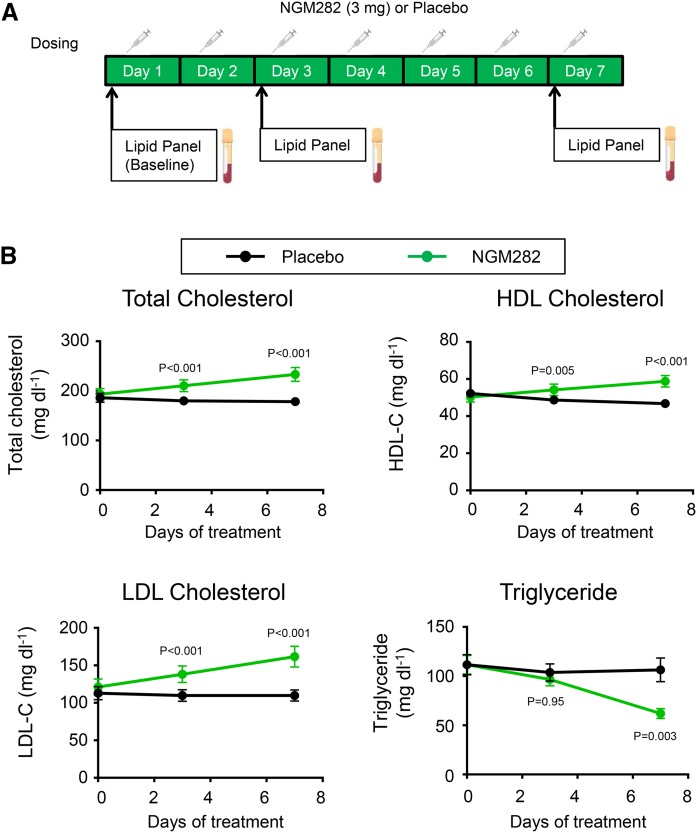
Effects of FGF19 analog NGM282 on lipid levels in healthy human subjects. A: Healthy volunteers were randomized to receive daily subcutaneous NGM282 protein (3 mg/day, n = 9) or placebo (n = 17) for 7 days. Blood sampling was performed before drug administration on days 1, 3, and 7. B: NGM282 increases serum concentrations of total cholesterol, HDL-C, and LDL-C, and decreases levels of triglycerides, in healthy human subjects. Data are least-squares mean ± SEM. The difference between NGM282 and placebo groups was analyzed using an ANCOVA model with treatment group as the factor and baseline values of the outcome as cofactor (SAS, version 9.4). Numbers on the graphs are *P* values versus the placebo group. Details on participants’ baseline characteristics, as well as percent changes in lipids from baseline to day 7, are included in supplemental Tables S6–S8.

We evaluated the effects of NGM282 on lipid levels in healthy human subjects in a randomized double-blind placebo-controlled clinical trial. NGM282 protein (3 mg/day, n = 9) or placebo (n = 17) was administered as a daily subcutaneous injection for seven consecutive days (**Fig. 6A**). Serum lipid levels were assessed before dosing on days 1, 3, and 7. The baseline characteristics of the participants are presented in supplemental Table S6.

Treatment with NGM282 significantly increased serum concentrations of total cholesterol, HDL-C, and LDL-C (Fig. 6B). At day 7, increases in total cholesterol of 48.8 mg/dl (*P* < 0.001), HDL-C of 13.5 ± 2.3 mg/dl (*P* < 0.001), and LDL-C of 41.4 ± 5.8 mg/dl (*P* < 0.001) were observed in NGM282-treated subjects compared with subjects receiving placebo (supplemental Table S7). In contrast, serum levels of triglycerides were markedly reduced in NGM282-treated subjects (−53.2 ± 9.7 mg/dl vs. placebo; *P* = 0.003; Fig. 6B). The NGM282-associated placebo-adjusted percent changes in lipid levels were as follows: total cholesterol, 25% (*P* < 0.001); HDL-C, 26% (*P* < 0.001); LDL-C, 38% (*P* = 0.01); and triglycerides, −38% (*P* = 0.002) (supplemental Table S8).

In conclusion, treatment of healthy human subjects with NGM282 for seven days in a randomized double-blind placebo-controlled trial resulted in elevations in serum cholesterol, HDL-C, LDL-C, and a reduction in serum triglyceride levels.

## DISCUSSION

In this report, we show that the endocrine hormone, FGF19, has a hitherto unsuspected intrinsic role in promoting transhepatic cholesterol efflux and HDL biogenesis. In particular, both FGF19 and its analog, NGM282, selectively activate LXR signaling pathways in the liver to enable anti-inflammatory and anti-atherosclerotic responses, while ameliorating hepatic steatosis. Furthermore, we show that administration of NGM282 to healthy volunteers for 7 days resulted in a 26% increase in HDL levels compared with placebo. Taken together, our data show that the effects of both WT and engineered forms of FGF19 on cholesterol metabolism are not limited to the inhibition of bile acid synthesis; rather, they involve unexpected complexity between multiple regulatory pathways.

The LXRs are nuclear receptors that bind oxysterols and activate a set of genes encoding proteins involved in reverse transport of cholesterol from peripheral tissues to the liver (20). LXR activation induces the expression of genes involved in cholesterol efflux, facilitates cholesterol esterification by promoting fatty acid synthesis, and exhibits anti-inflammatory effects through inhibition of TLR signaling (27). Mice lacking LXRs accumulate sterols in their tissues and manifest accelerated atherosclerosis; whereas, synthetic LXR agonists promote reverse cholesterol transport and protect mice against atherosclerosis (41, 42). Despite these potentially beneficial effects on cholesterol homeostasis, the development of LXR agonists as anti-atherogenic agents has been hindered by the massive accumulation of triglycerides (or hepatic steatosis) that accompanies activation of LXR in the liver (29, 43). In contrast, by selective modulation of LXR activity, FGF19 and NGM282 can overcome this hurdle while attenuating inflammation and protecting against atherosclerosis. Previous studies suggested that FGF19 does not directly activate LXR signaling in cultured hepatocytes (44). It is possible that the LXR activation by FGF19 and NGM282 may be a secondary consequence of reduced conversion of cholesterol to bile acids, which leads to a rise in intrahepatic cholesterol and oxysterol content, consistent with the known ability of cholesterol metabolites to activate LXR signaling (32). The marked net loss in liver fat by FGF19 and NGM282 is likely mediated by the FGFR1c-βKlotho and FGFR4-βKlotho receptor complexes, as an agonistic antibody against FGFR1c-βKlotho reduces steatosis (45) and a compound upregulating CYP7A1 increases steatosis (46).

Both FGF19 and NGM282 induce the hepatic expression of SCARB1, a protein that regulates HDL uptake by the liver and has demonstrated an anti-atherosclerotic effect when overexpressed in various mouse models (21, 47). However, despite the increase in HDL uptake associated with the elevated *Scarb1* levels, circulating concentrations of HDL actually rise in the presence of FGF19 and NGM282, as *Abca1* expression is also upregulated and the capacity for HDL biogenesis increases in parallel. Conditions of severe HDL deficiency and increased atherosclerosis have been described in some studies when the ABCA1 transporter is dysfunctional, including both in mice from which ABCA1 is ablated in the liver (48) as well as in patients with Tangier disease (49–51). Given the lack of expression of the FGFR-βKlotho receptor complex on macrophages, it appears that FGF19 and NGM282 may exert the anti-atherosclerotic effect through the direct or indirect induction of hepatic ABCA1. Thus, the coordinate upregulation of hepatic *Scarb1* and *Abca1* by FGF19 and NGM282 may synergistically act to stimulate reverse cholesterol transport.

We found that the FGF19 analog, NGM282, significantly increased circulating concentrations of LDL, in addition to HDL, in mice and in human subjects. The NGM282-associated increase in LDL-C concentrations was more pronounced than that observed for HDL-C in human subjects (+38% vs. +26% for LDL-C and HDL-C, respectively). Of note, larger increases in LDL-C than HDL-C were also observed with LXR agonists in human subjects (43). It appears that LXR agonism produces marked LDL-C elevations in species containing cholesteryl ester transfer protein (such as humans, but not mice), an effect not apparent from murine studies (52). While the elevation in HDL is encouraging, the rise in LDL, a major risk factor for cardiovascular disease (1), represents an important cause for concern. While LXR is reported to inhibit cholesterol uptake by the LDL receptor via Idol-dependent ubiquitination (53), the rise in serum LDL-C associated with NGM282 is unlikely attributed to the LDL receptor because NGM282 still increased LDL-C levels in mice deficient in LDLR. Mechanistically, we showed that the ablation of hepatic ABCA1 resulted in a profound reduction of serum HDL, as expected; however, the NGM282-associated LDL rise was entirely blunted in these mice. Whereas the pathways regulating the synthesis and uptake of LDL are well characterized, the molecular mechanisms regulating circulating HDL and LDL remain complex and are influenced by myriad factors. It is well-known that patients with Tangier’s disease (with genetic mutations or deficiency in ABCA1) have very low serum HDL. However, an important but often overlooked observation is that these patients also have low LDL levels (a 50% reduction) (54), suggesting that ABCA1 may also be involved in regulating circulating LDL. Similarly, mice with targeted disruption of ABCA1 had an almost complete absence of HDL, as expected, but levels of LDL were also markedly reduced (a 70% decrease) compared with WT littermates (55). Conversely, hepatic overexpression of ABCA1 via adenovirus raised plasma levels of total cholesterol and HDL, as well as LDL, in mice (56). Moreover, recent studies suggest that hepatic ABCA1 can reduce LDL catabolism and the production and secretion of VLDL triglyceride (57, 58). Therefore, contrary to previous assumptions, the role of ABCA1 in regulating circulating LDL seems under-appreciated. Our data provide evidence that hepatic ABCA1 can modulate the mobilization of LDL following NGM282 treatment, in addition to its well-established role in HDL biogenesis. In no way do these considerations diminish the need for LDL control; and as we have shown in this report, rosuvastatin or a neutralizing antibody against PCSK9 can effectively inhibit the FGF19-induced LDL increase in mice.

Both FGF19 and NGM282 increase the hepatic expression of ABCG5 and ABCG8, essential sterol transporters that mediate cholesterol excretion into bile to protect the liver against the accumulation of cholesterol (23). Inactivation of either ABCG5 or ABCG8 causes sitosterolemia, a genetic disorder characterized by sterol accumulation and premature coronary atherosclerosis. In recent years, ABCG5 and ABCG8 sterol transporters have been recognized as more than a defense against xenosterols. For example, mice deficient in ABCG5 and ABCG8 are prone to the development of fatty liver disease and liver injury (59). The induction of ABCG5 and ABCG8 may contribute to the anti-steatotic action of FGF19 and NGM282, although their impact on plasma cholesterol levels may be minimal (60).

NGM282 is not anticipated to exert direct actions on endothelial, immune, and smooth muscle cells that are involved in atherosclerotic plaque formation, due to the lack of receptor expression in these cell types. The inhibition of the bile acid synthetic pathway by NGM282 may indirectly contribute to the observed beneficial effects on atherosclerosis progression. Chronic exposure to high levels of bile acids can increase the expression of ICAM-1/VCAM-1 and promote the adhesion of monocytes to endothelium, thereby contributing to the initiation of vascular lesion formation (61). Bile acids are also known to exert direct regulatory functions on macrophages and smooth muscle cells that are central to the development of atherosclerotic lesions in vessel walls (62). Suppression of bile acid synthesis by NGM282 may impact favorably on endothelial function. Therefore, the mechanisms by which NGM282 protects against atherosclerosis may be multifactorial and are not limited to the selective activation of hepatic LXR, the promotion of HDL biogenesis via ABCA1, and the associated impact on reverse cholesterol transport and systemic inflammatory status.

There are several limitations in this study. We did not establish the requirement of LXR in the observed phenotypes. We also did not study the role of LRH-1 and HNF4α, transcription factors crucial in cholesterol homeostasis, in lipid changes associated with FGF19 and NGM282. Recent studies have demonstrated that it is the ability of hepatic LXR activation to increase HDL biogenesis that is largely responsible for the enhanced reverse cholesterol transport and the anti-atherogenic effect of LXR agonists (63). Nevertheless, mice with LXR deficiency in hepatocytes are needed to provide a better understanding of the molecular mechanisms of the NGM282 therapy.

The development of novel therapies to exploit the atheroprotective property of HDL has been an area of intense investigation in recent years, but has proven to be particularly challenging (5, 6). Our results suggest that the FGF19 analog, NGM282, may represent an alternative strategy by coordinately enhancing HDL biogenesis and transhepatic cholesterol efflux, while ameliorating steatosis, and providing protection against the development of atherosclerotic plaques. Of note, serum FGF19 concentration positively correlates with HDL-C levels, and lower FGF19 concentrations are associated with both the presence and severity of coronary artery diseases in humans (40). Overall, this report reveals a previously undescribed role of FGF19 on cholesterol metabolism and offers a differentiated mechanism for the control of atherosclerosis progression. However, long-term safety and efficacy studies through clinical trials will be required to fully assess the risks and benefits of therapeutic FGF19 analogs.

## Supplementary Material

Supplemental Data
