# Supplementary material for: Therapeutic FGF19 promotes HDL biogenesis and transhepatic cholesterol efflux to prevent atherosclerosis
Source: J Lipid Res. 2019 Jan 24;60(3):550–65. doi: 10.1194/jlr.M089961 (PMC6399511; doi:10.1194/jlr.M089961)
Supplement: Supplemental Data [file supp_60_3_550__index.html]

Therapeutic FGF19 promotes HDL biogenesis and transhepatic cholesterol efflux to prevent atherosclerosis — Therapeutic FGF19 promotes HDL biogenesis and transhepatic cholesterol efflux to prevent atherosclerosis — Supplemental Data 

# Therapeutic FGF19 promotes HDL biogenesis and transhepatic cholesterol efflux to prevent atherosclerosis

## Supplemental Data

- supplemental file (.pdf, 2.5 MB) - Supplemental file with highlights removed
