# Supplementary material for: Therapeutic FGF19 promotes HDL biogenesis and transhepatic cholesterol efflux to prevent atherosclerosis
Source: J Lipid Res. 2019 Jan 24;60(3):550–65. doi: 10.1194/jlr.M089961 (PMC6399511; doi:10.1194/jlr.M089961)
Supplement: Supplemental Data [file 10.1194_M089961_jlr.M089961-1.pdf]

SUPPLEMENTARY INFORMATION

Therapeutic FGF19 promotes HDL biogenesis and transhepatic cholesterol efflux to prevent atherosclerosis

Mei Zhou, R. Marc Learned, Stephen J. Rossi, Hui Tian, Alex M. DePaoli and Lei Ling*

NGM Biopharmaceuticals, Inc.

333 Oyster Point Blvd. South San Francisco, CA 94080

*Corresponding author: Lei Ling, NGM Biopharmaceuticals, Inc., 333 Oyster Point Blvd. South San Francisco, CA 94080, Tel: 650-243-5546, Email: lling@ngmbio.com

SUPPLEMENTARY INFORMATION

SUPPLEMENTARY INFORMATION 2

Supplemental Figures 3

Supplementary Figure S1. Genes in canonical pathways that are regulated by FGF19 in *db/db* mice 3

Supplementary Figure S2. Levels of intrahepatic cholesterol and hydroxycholesterol in *db/db* mice treated with FGF19 and NGM282. 4

Supplementary Figure S3. Lack of activation of LXR target genes in the ileum by FGF19 and NGM282. 5

Supplementary Figure S4. The anti-steatotic effect of NGM282 occurs independent of hepatocellular ABCA1 and ABCG1...6

Supplementary Figure S5. FGFR4-deficient mice are resistant to FGF19-induced hepatocarcinogenesis. 7

Supplementary Figure S6. FGF19-associated cholesterol changes can be inhibited by rosuvastatin or a neutralizing antibody against PCSK9...8

Supplementary Figure S7. LDL receptor is dispensable for NGM282-associated cholesterol change. 9

Supplementary Figure S8. FGF19 analogue NGM282 protects against atherosclerosis in *Apoe*$^{-/-}$ mice fed a Western diet without affecting circulating cholesterol levels...10

Supplemental Tables...11

Supplementary Table S1. Top canonical pathways affected by FGF19 treatment in *db/db* mice 11

Supplementary Table S2. Regulation of genes in LXR/RXR signaling by FGF19 in *db/db* mice..........................12

Supplementary Table S3. GTEx datasets used in the current study 13

Supplementary Table S4. Datasets on patients with cardiovascular disease used in the current study 14

Supplementary Table S5. Downregulation of FGF19 in patients with cardiovascular disease 15

Supplementary Table S6. Baseline participants' characteristics in a double-blind, placebo-controlled trial of NGM282 in healthy human subjects 16

Supplementary Table S7. Change from baseline to day 7 in serum lipids in participants treated with NGM282...17

Supplementary Table S8. Percent change from baseline to day 7 in serum lipids in participants treated with NGM282 18

Supplementary Table S9. Clinical chemistry reagents used in the study 19

Supplementary Table S10. Primers for quantitative real-time PCR analysis 20

Supplemental Figures

Atherosclerosis signaling

Gene symbol	Log2 ratio (FGF19/control)	Intensity
ALOX15	↓-1.402	48.719
APOA4*	↑1.041	137.819
APOM	↑1.853	920.874
CCR2	↓-3.175	12.520
CCR3	↓-1.344	11.299
COL1A1	↓-2.254	87.349
COL1A2	↓-2.137	81.524
COL3A1	↓-2.094	95.131
CXCR4	↓-1.817	38.313
ICAM1	↓-1.947	99.777
IL1A	↓-1.846	38.803
IL1B	↓-3.024	20.315
ITGA4	↓-2.800	39.688
ITGB2	↓-2.867	89.548
LPL	↓-1.863	207.967
LYZ*	↓-2.623	538.903
MSR1	↓-1.635	165.377
PDGFC	↓-1.347	60.940
PLA2G7	↓-1.569	291.515
PLA2G16	↓-1.481	49.016
PLA2G4A	↓-2.405	33.354
PLA2G4F	↑0.760	25.144
PNPLA3	↑4.014	683.804
SELPLG	↓-1.450	33.046
TNF	↓-1.699	12.694
TNFRSF14	↓-1.332	100.260
VCAM1	↓-2.067	117.184

LXR/RXR activation

Gene symbol	Log2 ratio (FGF19/control)	Intensity
A1BG	↓-3.219	26.994
ABCG5	↑1.342	2845.524
ABCG8	↑1.492	1521.306
ACACA*	↑1.728	932.528
APOA4*	↑1.041	137.819
APOM	↑1.853	920.874
CYP7A1	↓-3.540	94.352
FASN	↑1.793	4334.070
HMGCR	↑0.998	1670.552
IL1A	↓-1.846	38.803
IL1B	↓-3.024	20.315
IL1RAP	↑0.821	1013.782
LPL	↓-1.863	207.967
LYZ*	↓-2.623	538.903
MLXIPL	↑0.942	936.122
MSR1	↓-1.635	165.377
NOS2	↓-3.271	18.007
PTGS2	↓-2.617	15.381
SAA1	↓-3.921	263.416
SCD	↑0.845	11118.271
SREBF1	↑1.485	1188.462
TLR4	↓-1.666	18.777
TNF	↓-1.699	12.694

FXR/RXR activation

Gene symbol	Log2 ratio (FGF19/control)	Intensity
A1BG	↓-3.219	26.994
ABCG5	↑1.342	2845.524
ABCG8	↑1.492	1521.306
AKT3	↓-1.361	30.484
APOA4*	↑1.041	137.819
APOM	↑1.853	920.874
CYP7A1	↓-3.540	94.352
CYP8B1	↓-1.317	763.160
FASN	↑1.793	4334.070
FOXA3	↑0.809	385.947
IL1A	↓-1.846	38.803
IL1B	↓-3.024	20.315
LPL	↓-1.863	207.967
MLXIPL	↑0.942	936.122
PKLR	↑1.177	4291.831
SAA1	↓-3.921	263.416
SCARB1	↑0.765	1622.121
SLC22A7	↑1.368	292.978
SREBF1	↑1.485	1188.462
SULT2A1	↑2.137	50.951
TNF	↓-1.699	12.694

Pattern recognition receptors in recognition of bacteria and viruses

Gene symbol	Log2 ratio (FGF19/control)	Intensity
C1QA	↓-1.711	272.875
C1QB	↓-1.855	289.775
C1QC	↓-1.760	659.633
C3AR1	↓-1.787	47.734
C5AR1	↓-1.669	23.118
CASP1	↓-1.935	22.623
CCL5	↓-3.225	138.396
CLEC6A	↓-2.919	75.738
CLEC7A	↓-3.255	52.780
IL1A	↓-1.846	38.803
IL1B	↓-3.024	20.315
PRKCB	↓-1.452	35.550
PRKCD	↓-1.933	104.607
SYK	↓-1.303	40.419
TLR1	↓-1.724	17.768
TLR2	↓-1.522	51.605
TLR4	↓-1.666	18.777
TLR7	↓-1.420	18.122
TLR8	↓-1.513	11.907
TNF	↓-1.699	12.694

Th1 and Th2 activation pathway

Gene symbol	Log2 ratio (FGF19/control)	Intensity
CCR1	↓-2.146	7.726
CCR3	↓-1.344	11.299
CD80	↓-1.376	12.161
CD86	↓-2.158	28.533
CD274	↓-3.116	50.884
CD3E	↓-1.312	32.806
CD3G	↓-3.044	23.627
CD8A	↓-1.976	42.908
CXCR4	↓-1.817	38.313
CXCR6	↓-1.966	15.489
HLA-DMA	↓-2.649	32.904
HLA-DQA1	↓-3.222	209.372
HLA-DQB1	↓-3.499	317.977
HLA-DRA	↓-3.268	273.462
HLA-DRB5	↓-3.375	237.474
ICAM1	↓-1.947	99.777
ICOS	↓-1.486	20.210
IKZF1	↓-1.768	21.352
IL10RA	↓-1.991	35.427
IL2RB	↓-2.162	23.510
IL2RG	↓-3.433	34.329
IRF1	↓-1.947	326.727
ITGB2	↓-2.867	89.548
KLRC1	↓-2.215	8.316
MAP2K6	↑1.148	127.257
NFIL3	↓-1.347	112.981
SPI1	↓-1.996	51.094

Dendritic cell maturation

Gene symbol	Log2 ratio (FGF19/control)	Intensity
AKT3	↓-1.361	30.484
CD80	↓-1.376	12.161
CD86	↓-2.158	28.533
COL1A1	↓-2.254	87.349
COL1A2	↓-2.137	81.524
COL3A1	↓-2.094	95.131
FCER1G	↓-2.415	244.672
FCGR1A	↓-2.017	42.291
FCGR2A	↓-2.011	57.388
FCGR3A/FCGR3B	↓-2.589	133.778
HLA-DMA	↓-2.649	32.904
HLA-DQA1	↓-3.222	209.372
HLA-DQB1	↓-3.499	317.977
HLA-DRA	↓-3.268	273.462
HLA-DRB5	↓-3.375	237.474
ICAM1	↓-1.947	99.777
IL1A	↓-1.846	38.803
IL1B	↓-3.024	20.315
IRF8	↓-2.235	72.571
LEPR	↑2.244	111.307
STAT1	↓-1.743	907.382
TLR2	↓-1.522	51.605
TLR4	↓-1.666	18.777
TNF	↓-1.699	12.694
TYROBP	↓-1.969	166.484

.

Supplementary Figure S1. Genes in canonical pathways that are regulated by FGF19 in *db/db* mice
RNA was extracted from livers of *db/db* mice 2 weeks after intravenous injection of AAV-FGF19, or a control virus carrying green fluorescent protein. Genes differentially regulated by FGF19 from transcriptome profiling were examined by Ingenuity pathway analysis. Green arrows indicate downregulation (FGF19 vs. control), red arrows indicate upregulation (FGF19 vs. control).

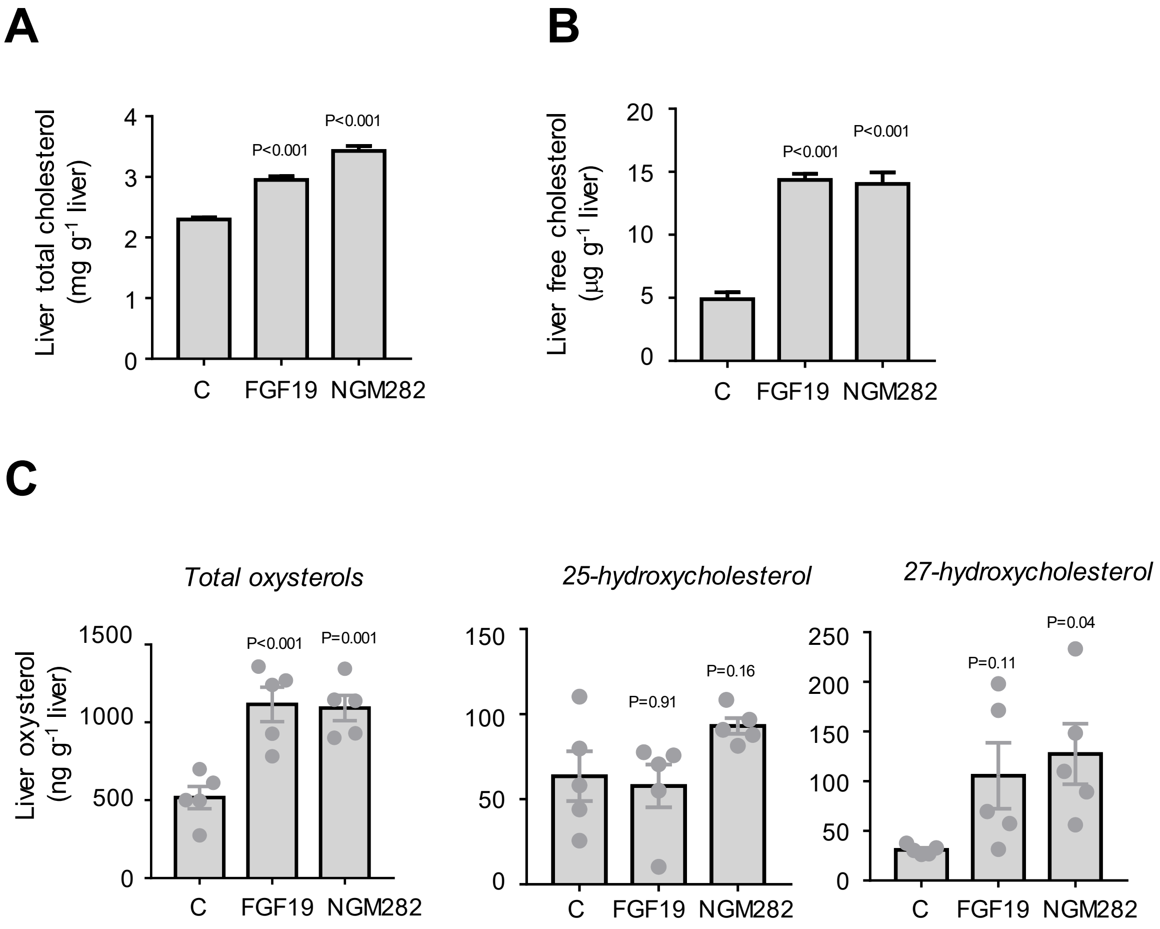

Supplementary Figure S2. Levels of intrahepatic cholesterol and hydroxycholesterol in *db/db* mice treated with FGF19 and NGM282.
A. Hepatic total cholesterol content. Lipids were extracted from livers of *db/db* mice 2 weeks after intravenous injection of AAV-FGF19, AAV-NGM282 or a control (C) virus carrying green fluorescent protein. Concentrations of intrahepatic total cholesterol were measured using Infinity Liquid Stable Reagents (Thermo Fisher). n=5 mice per group. **B.** Intrahepatic free cholesterol content. n=5 mice per group. **C.** Intrahepatic hydroxycholesterol content. Intrahepatic free cholesterol and hydroxycholesterol were analyzed on a Michrom Paradigm HPLC equipped with a C18AQ analytical column (2.0×150 mm, 4 µm), auto sampler, helium degassing system and a column oven. Column eluent was introduced to a LTQ-Orbitrap Velos mass spectrometer (Thermo Fisher) with a heated electrospray ionization source. n=5 mice per group. Data are mean±s.e.m., numbers on the graphs are P values vs the control group by one-way ANOVA with Dunnett's post test.

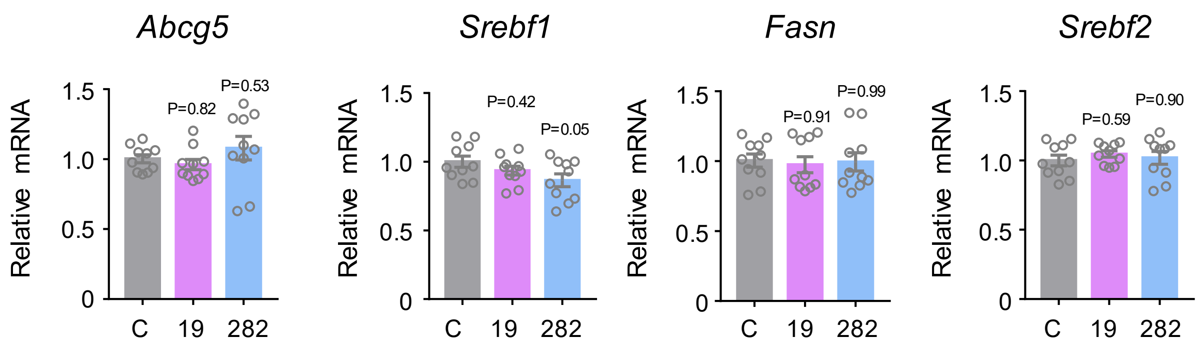

Supplementary Figure S3. Lack of activation of LXR target genes in the ileum by FGF19 and NGM282. Ileum was harvested from *db/db* mice 2 weeks after intravenous injection of AAV-FGF19 (19), AAV-NGM282 (282), or a control virus (C). *n*=10 biologically independent samples per group. RNA was extracted from ileum for qPCR analysis of LXR target genes (*Abcg5, Srebf1, Fasn, and Srebf2*). Data are mean±s.e.m., numbers on the graphs are P values vs the control group by one-way ANOVA with Dunnett's post test.

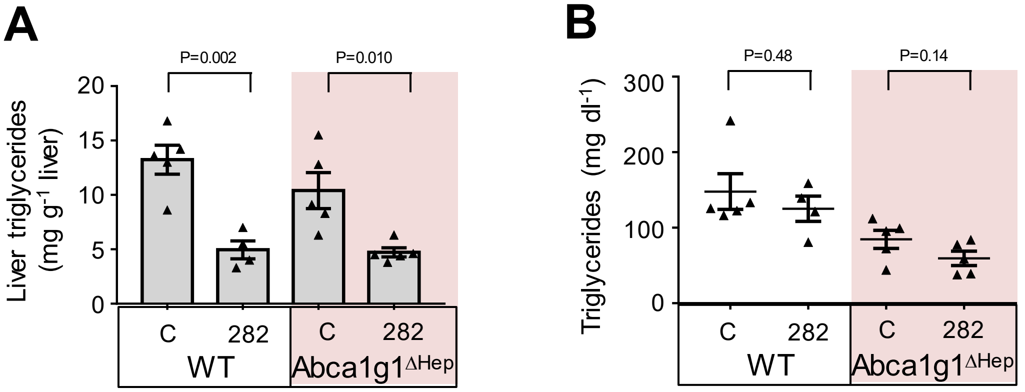

Supplementary Figure S4. The anti-steatotic effect of NGM282 occurs independent of hepatocellular ABCA1 and ABCG1.

A. Hepatic triglyceride content by Folch method. Concentrations of intrahepatic triglycerides were measured using Infinity Liquid Stable Reagents (Thermo Fisher). Values were expressed as milligrams of triglycerides per gram wet weight of liver. Wild type (WT) mice treated with control (C) virus, *n*=5 mice; WT mice treated with NGM282, *n*=4 mice; *Abca1g1*$^{\Delta Hep}$ mice treated with control virus, *n*=5 mice; *Abca1g1*$^{\Delta Hep}$ mice treated with NGM282, *n*=5 mice.

B. Serum concentrations of triglycerides. Serum samples were collected 4 weeks after intravenous injection of AAV-NGM282, or a control (C) virus. Wild type (WT) mice treated with control virus, *n*=5 mice; WT mice treated with NGM282, *n*=4 mice; *Abca1g1*$^{\Delta Hep}$ mice treated with control virus, *n*=5 mice; *Abca1g1*$^{\Delta Hep}$ mice treated with NGM282, *n*=5 mice. Data are mean±s.e.m., numbers on the graphs are P values vs the control group by unpaired, two-tailed, *t*-test.

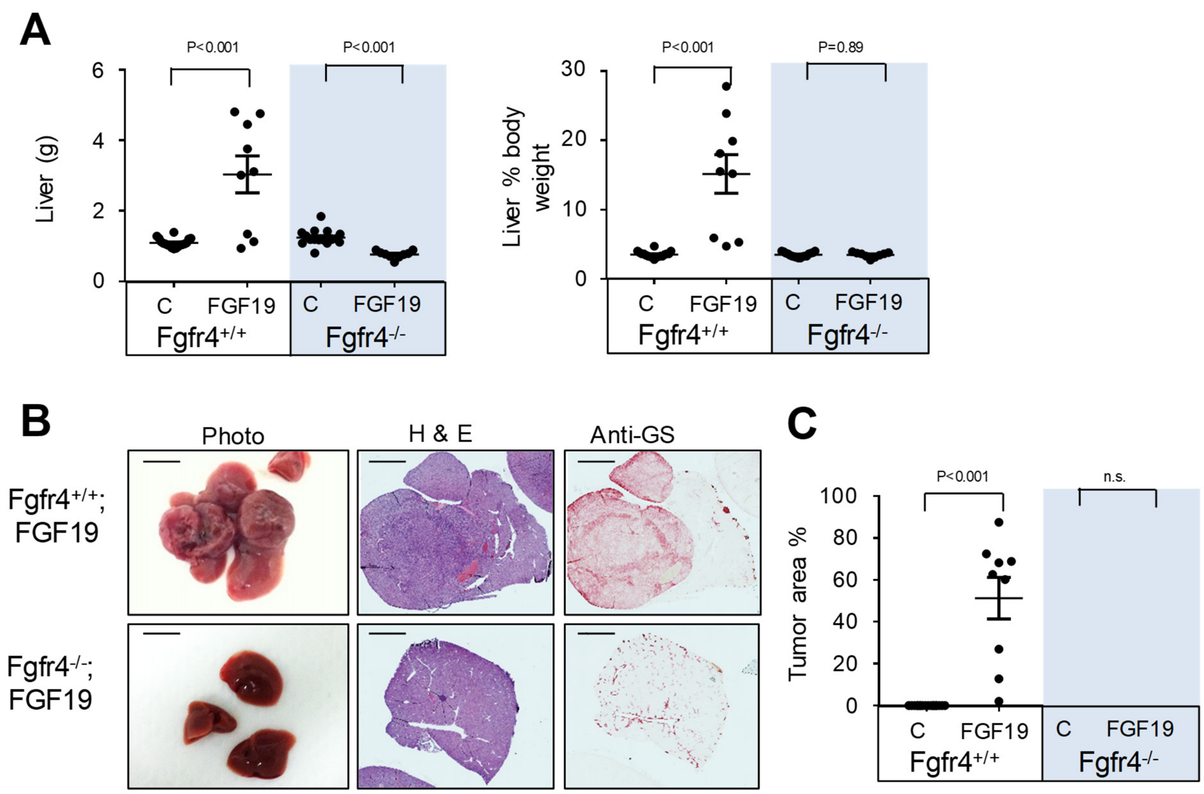

Supplementary Figure S5. FGFR4-deficient mice are resistant to FGF19-induced hepatocarcinogenesis. *Fgfr4*$^{+/+}$ or *Fgfr4*$^{-/-}$ mice were intravenously injected with AAV-FGF19 or a control (C) virus. Mice were sacrificed 12 months later for analysis of serum cholesterol, body weight, liver weight and liver tumors. *Fgfr4*$^{+/+}$ mice injected with control virus, *n*=18 mice; *Fgfr4*$^{+/+}$ mice injected with AAV-FGF19, *n*=9 mice; *Fgfr4*$^{-/-}$ mice injected with control virus, *n*=17 mice; *Fgfr4*$^{-/-}$ mice injected with AAV-FGF19, *n*=9 mice. Data presented are from one of three independent experiments with similar results. **A.** FGFR4 deficiency abolishes FGF19-associated increases in liver weight and ratios of liver-to-body weight. **B**. Representative images of macroscopic view, and liver sections stained with H&E or anti-glutamine synthetase (GS). Vector Red substrates (red color) were used for immunohistochemistry. Scale bars, 5 mm. **C**. Quantification of glutamine synthetase-positive tumor area as a percentage of total liver area. Images for the entire liver section was acquired using Surveyor program. For morphometric analysis of tumor area, glutamine synthetase-positive tumor areas were quantified using Measure/Count/Area tool from ImagePro software. Data are mean±s.e.m., numbers on the graphs are *P* values *vs* the control group by unpaired, two-tailed, *t*-test. *n.s.,* not significant.

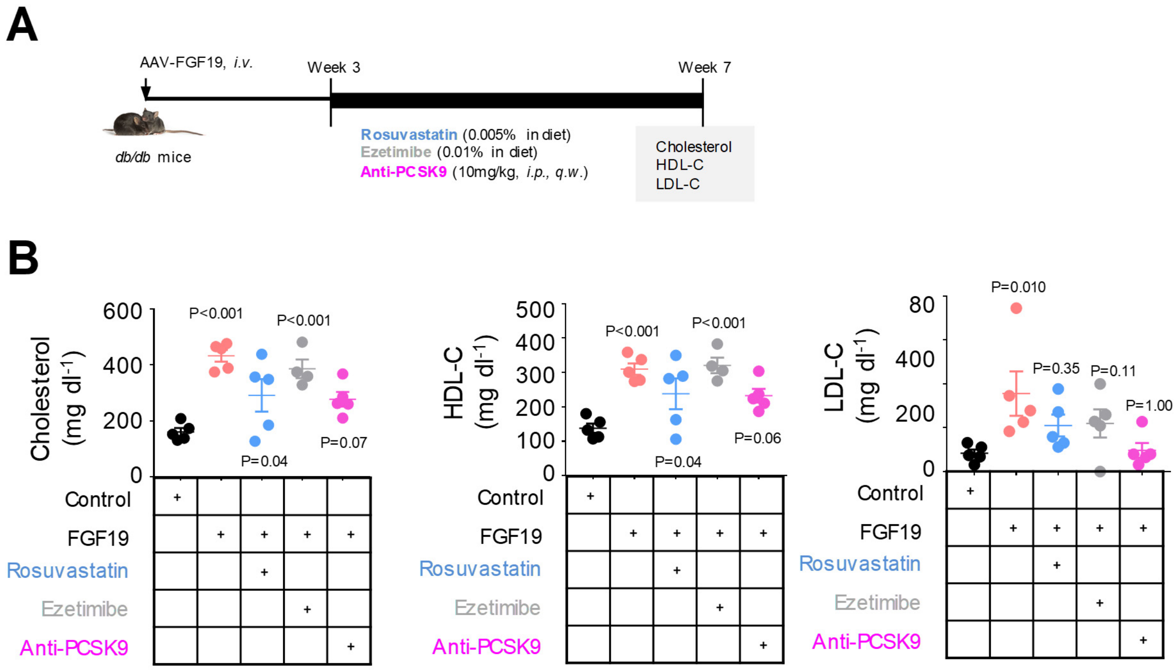

Supplementary Figure S6. FGF19-associated cholesterol changes can be inhibited by rosuvastatin or a neutralizing antibody against PCSK9.

A. Study design. *db/db* mice were administered with AAV-FGF19 via tail vein. Three weeks later, mice were treated with rosuvastatin (0.005% in diet, *n*=5 mice), ezetimibe (0.01% in diet, *n*=4 mice), or anti-PCSK9 neutralizing antibody (10 mg kg^{-1}, *i.p., q.w.*, *n*=5 mice) for an additional 4 weeks. *db/db* mice administered with AAV-FGF19 (*n*=5 mice) or a control virus (*n*=5 mice) on regular chow diet were also included. **B**. Serum levels of total cholesterol, HDL-C and LDL-C. Data are mean±s.e.m., numbers on the graphs are *P* values *vs* the control group by one-way ANOVA with Dunnett's post test.

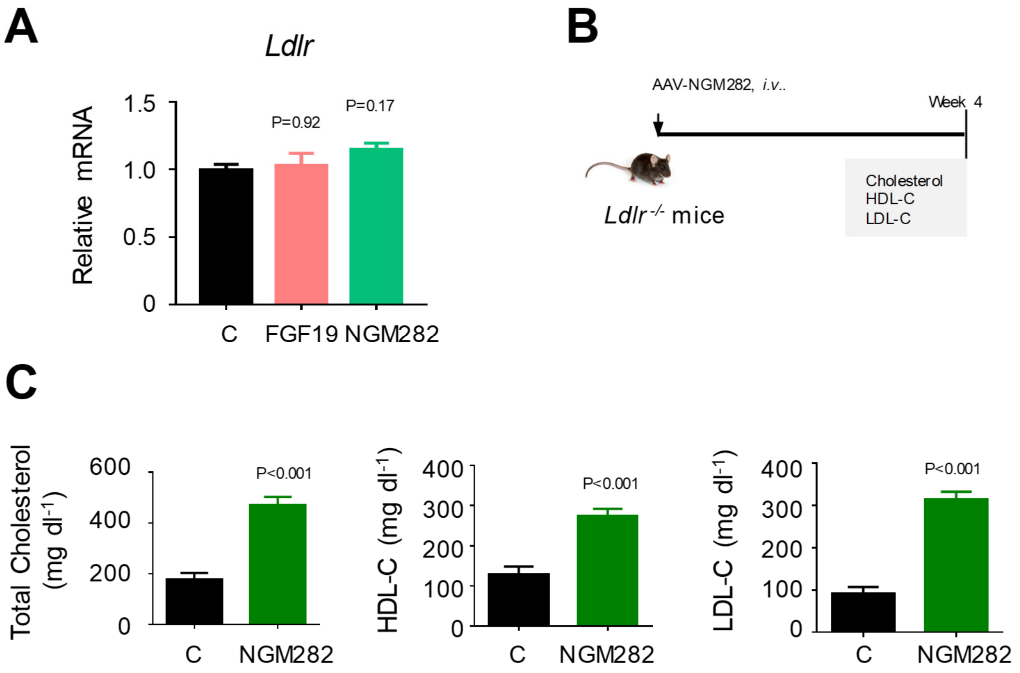

Supplementary Figure S7. LDL receptor is dispensable for NGM282-associated cholesterol change.
A. qPCR analysis of mRNA levels of LDLR. Livers were harvested from *db/db* mice 2 weeks after intravenous injection of AAV-FGF19, AAV-NGM282, or a control (C) virus. n=10 biologically independent samples per group. **B**. Study design in *Ldlr*$^{-/-}$ mice. *Ldlr*$^{-/-}$ mice were administered with AAV-NGM282 or a control virus via tail vein and analyzed 4 weeks later. **C**. Serum levels of total cholesterol, HDL-C and LDL-C 4 weeks after administration of AAV-NGM282 or a control virus in *Ldlr*$^{-/-}$ mice (n=5 mice per group). Data are mean±s.e.m., numbers on the graphs are P values *vs* the control group by one-way ANOVA with Dunnett's post test for multi-group comparison or unpaired, two-tailed, t-test.

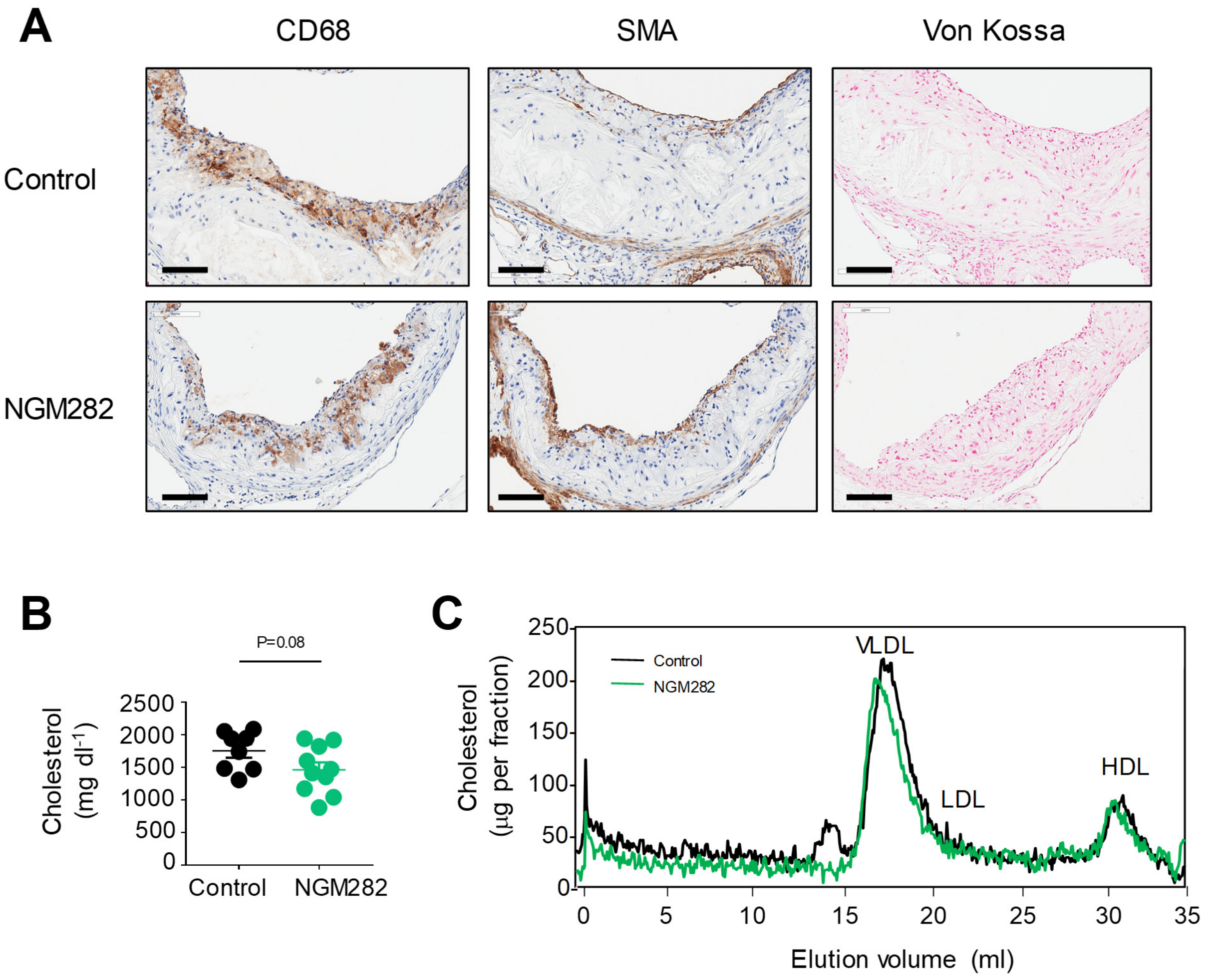

Supplementary Figure S8. FGF19 analogue NGM282 protects against atherosclerosis in *Apoe*$^{-/-}$ mice fed a Western diet without affecting circulating cholesterol levels.

A. Representative images of atherosclerotic plaque in cross sections of the aortic root area. Sections were stained with anti-CD68 (for macrophage), anti-smooth muscle actin (for smooth muscle cells), or von Kossa (for calcium) as indicated. Visualization with DAB substrate is shown as brown color. Scale bars, 100 µm. *Apoe*$^{-/-}$ mice received intravenous injection of AAV-NGM282 or a control virus, and were fed a high-fat (21% *w/w* fat), high-cholesterol (0.15% *w/w* cholesterol) Western diet for 18 weeks. *Apoe*$^{-/-}$ mice injected with control virus, *n*=8 mice; *Apoe*$^{-/-}$ mice injected with AAV- NGM282, *n*=10 mice. **B**. Serum total cholesterol levels of *Apoe*$^{-/-}$ mice treated with NGM282 or a control virus. *Apoe*$^{-/-}$ mice injected with control virus, *n*=8 mice; *Apoe*$^{-/-}$ mice injected with AAV- NGM282, *n*=10 mice. Data are mean±s.e.m., numbers on the graphs are *P* values *vs* the control group by unpaired, two-tailed, *t*-test. **C**. Fractionation of circulating lipids by fast protein liquid chromatography (FPLC). Pooled mouse serum samples were injected on two Superose 6 HR 10/30 columns connected in series on AKTA Explorer FPLC system. Lipoproteins were eluted at a constant 0.3 ml min^{-1} flow rate with phosphate-buffered saline (pH 7.4) containing 0.02% EDTA. Individual fractions were collected for total cholesterol measurements. *Apoe*$^{-/-}$ mice injected with control virus, pooled serum from *n*=8 mice; *Apoe*$^{-/-}$ mice injected with AAV-NGM282, pooled serum from *n*=10 mice. VLDL, very low-density lipoprotein.

Supplemental Tables

Supplementary Table S1. Top canonical pathways affected by FGF19 treatment in *db/db* mice

Ingenuity Canonical Pathways	-Log (P-value)	Z-score	Molecules in Pathway
Atherosclerosis Signaling	15.3	#	CCR3,IL1A,ICAM1,APOA4,MSR1,CCR2,PLA2G7,PDGFC,COL1A2,LYZ,LPL,PNPLA3,PLA2G4F,ITGA4,PLA2G16,ALOX15,VCAM1,APOM,CXCR4,TNFRSF14,SELPLG,PLA2G4A,ITGB2,COL1A1,IL1B,TNF,COL3A1
Th1 and Th2 Activation Pathway	13.6	#	MAP2K6,CCR3,ICAM1,CD3E,HLA-DQA1,HLA-DQB1,CD8A,SPI1,NFIL3,HLA-DMA,HLA-DRA,CD274,STAT1,TIMD4,IL2RB,KLRC1,CCR1,IL2RG,CXCR4,IKZF1,IRF1,ITGB2,CD3G,CD80,ICOS,CXCR6,IL10RA,CD86,VAV1,HLA-DRB5
LXR/RXR Activation	12.1	1.732	ABCG8,SCD,IL1A,MLXIPL,ABCG5,APOA4,APOM,MSR1,A1BG,TLR4,LYZ,SREBF1,FASN,SAA1,LPL,CYP7A1,IL1B,ACACA,PTGS2,NOS2,HMGCR,IL1RAP,TNF
Granulocyte Adhesion and Diapedesis	11.7	#	IL1A,SELL,ICAM1,CCL24,SDC3,CCL5,Cxcl9,CXCL10,CCL3L3,Ccl2,MMP12,Ccl6,IL1RAP,ITGA4,VCAM1,C5AR1,CXCR4,ITGAL,FPR1,SELPLG,ITGB2,ITGAM,CCL4,CLDN1,IL1B,TNF,MSN
Communication between Innate and Adaptive Immune Cells	11	#	IL1A,TLR8,CCL5,CD8A,TLR2,CXCL10,TLR4,CCL4,CD80,CCL3L3,TLR1,HLA-DRA,TLR7,FCER1G,CD86,Tlr13,IL1B,TNF,HLA-DRB5
FXR/RXR Activation	9.95	#	ABCG8,IL1A,MLXIPL,ABCG5,APOM,APOA4,PKLR,SLC22A7,A1BG,SULT2A1,CYP8B1,SCARB1,SREBF1,SAA1,FASN,LPL,CYP7A1,AKT3,IL1B,TNF,FOXA3
Dendritic Cell Maturation	9.43	-4.379	IL1A,ICAM1,LEPR,HLA-DQA1,HLA-DQB1,FCGR1A,COL1A2,HLA-DMA,HLA-DRA,AKT3,STAT1,FCGR3A/FCGR3B,TYROBP,FCGR2A,TLR2,TLR4,COL1A1,CD80,FCER1G,CD86,IL1B,IRF8,TNF,HLA-DRB5,COL3A1
Th1 Pathway	9.37	-2.84	MAP2K6,ICAM1,CD3E,HLA-DQA1,HLA-DQB1,CD8A,IRF1,CD3G,ITGB2,NFIL3,CD80,HLA-DMA,ICOS,HLA-DRA,IL10RA,CD86,CD274,VAV1,STAT1,HLA-DRB5,KLRC1
TREM1 Signaling	9.37	-4	ICAM1,TYROBP,CIITA,TLR8,LAT2,TLR2,TLR4,TLR1,TLR7,CASP1,CD86,AKT3,IL1B,Tlr13,TNF,ITGAX
Th2 Pathway	9.29	-2.887	CCR1,CCR3,IL2RG,ICAM1,CD3E,CXCR4,IKZF1,HLA-DQA1,HLA-DQB1,SPI1,CD3G,ITGB2,CD80,HLA-DMA,ICOS,HLA-DRA,CXCR6,CD86,VAV1,TIMD4,HLA-DRB5,IL2RB
Altered T Cell and B Cell Signaling in Rheumatoid Arthritis	9.05	#	IL1A,HLA-DQA1,TLR8,HLA-DQB1,TLR2,TLR4,CD80,HLA-DMA,TLR1,HLA-DRA,TLR7,FCER1G,CD86,Tlr13,IL1B,TNF,HLA-DRB5
Fcγ Receptor-mediated Phagocytosis in Macrophages and Monocytes	8.81	-4.123	RAC2,FCGR2A,FYB,FCGR1A,INPP5D,PLD4,HMOX1,NCF1,ACTA2,SYK,PRKCD,HCK,AKT3,VAV1,FCGR3A/FCGR3B,LCP2,PRKCB
T Helper Cell Differentiation	8.59	#	IL2RG,IL21R,HLA-DQA1,HLA-DQB1,RORC,CD80,HLA-DMA,ICOS,HLA-DRA,FCER1G,IL10RA,CD86,STAT1,TNF,HLA-DRB5
Leukocyte Extravasation Signaling	8.51	-3.838	RAC2,ICAM1,TIMP1,CYBA,CYBB,MMP12,ITGA4,VCAM1,CXCR4,ARHGAP4,NCF4,ITGAL,SELPLG,ITGB2,NCF1,ITGAM,ACTA2,CLDN1,RASGRP1,PRKCD,NCF2,CD44,VAV1,PRKCB,MSN
Role of Pattern Recognition Receptors in Recognition of Bacteria and Viruses	8.46	-3.873	IL1A,C5AR1,TLR8,C1QA,C1QC,CCL5,C1QB,TLR2,TLR4,CLEC7A,SYK,PRKCD,TLR1,TLR7,CASP1,IL1B,CLEC6A,TNF,C3AR1,PRKCB
Agranulocyte Adhesion and Diapedesis	8.07	#	IL1A,VCAM1,SELL,ICAM1,C5AR1,CXCR4,CCL24,CCL5,Cxcl9,SELPLG,CXCL10,ITGB2,CCL4,ACTA2,CCL3L3,CLDN1,Ccl2,IL1B,MMP12,TNF,Ccl6,ITGA4,MSN
iCOS-iCOSL Signaling in T Helper Cells	7.75	-3.207	IL2RG,CD3E,HLA-DQA1,HLA-DQB1,INPP5D,PTPRC,CD3G,CD80,HLA-DMA,ICOS,HLA-DRA,FCER1G,AKT3,VAV1,PLEKHA2,HLA-DRB5,LCP2,IL2RB

Ingenuity pathway analysis of genes differentially regulated in the livers from *db/db* mice treated with AAV-FGF19 or a control virus. Enriched canonical pathways are ranked by –Log (P value). Z-score > 0 indicates upregulated pathway; Z-score < 0 indicates downregulated pathways; Z-score #, no activity pattern available.

Supplementary Table S2. Regulation of genes in LXR/RXR signaling by FGF19 in *db/db* mice

Symbol	Entrez Gene Name	Log2 Ratio	Intensity/RPKM/FPKM/Counts	Entrez Gene ID for Human	Entrez Gene ID for Mouse
A1BG	alpha-1-B glycoprotein	-3.219	26.994	1	117586
ABCG5	ATP binding cassette subfamily G member 5	1.342	2845.524	64240	27409
ABCG8	ATP binding cassette subfamily G member 8	1.492	1521.306	64241	67470
ACACA	acetyl-CoA carboxylase alpha	1.728	932.528	31	107476
APOA4	apolipoprotein A4	1.041	137.819	337	11808
APOM	apolipoprotein M	1.853	920.874	55937	55938
CYP7A1	cytochrome P450 family 7 subfamily A member 1	-3.54	94.352	1581	13122
FASN	fatty acid synthase	1.793	4334.07	2194	14104
HMGCR	3-hydroxy-3-methylglutaryl-CoA reductase	0.998	1670.552	3156	15357
IL1A	interleukin 1 alpha	-1.846	38.803	3552	16175
IL1B	interleukin 1 beta	-3.024	20.315	3553	16176
IL1RAP	interleukin 1 receptor accessory protein	0.821	1013.782	3556	16180
LPL	lipoprotein lipase	-1.863	207.967	4023	16956
LYZ	lysozyme	-2.623	538.903	4069	17110|17105
MLXIPL	MLX interacting protein like	0.942	936.122	51085	58805
MSR1	macrophage scavenger receptor 1	-1.635	165.377	4481	20288
NOS2	nitric oxide synthase 2	-3.271	18.007	4843	18126
PTGS2	prostaglandin-endoperoxide synthase 2	-2.617	15.381	5743	19225
SAA1	serum amyloid A1	-3.921	263.416	6288	20209|20208
SCD	stearoyl-CoA desaturase	0.845	11118.27	6319	20249
SREBF1	sterol regulatory element binding transcription factor 1	1.485	1188.462	6720	20787
TLR4	toll like receptor 4	-1.666	18.777	7099	21898
TNF	tumor necrosis factor	-1.699	12.694	7124	21926

Ingenuity pathway analysis of genes in LXR/RXR signaling that are differentially regulated in the livers from *db/db* mice treated with AAV-FGF19 or a control virus. Negative Log2 ratios indicate downregulation by FGF19 treatment. Positive Log2 ratios indicate upregulation by FGF19 treatment.

Supplementary Table S3. GTEx datasets used in the current study

Tissue Type	Number of Patient Samples
Adipose	647
Adrenal gland	159
Bladder	11
Blood	540
Blood vessel	822
Brain	1609
Breast	218
Cervix uteri	11
Colon	376
Esophagus	788
Fallopian tube	7
Heart	547
Kidney	36
Liver	136
Lung	442
Muscle	557
Nerve	389
Ovary	108
Pancreas	193
Pituitary	125
Prostate	120
Salivary gland	70
Skin	725
Small intestine	104
Spleen	118
Stomach	203
Testis	199
Thyroid	424
Uterus	90
Vagina	97

Supplementary Table S4. Datasets on patients with cardiovascular disease used in the current study

Accession	Disease	PubMed
GSE1145	heart failure	n/a
GSE17800	dilated cardiomyopathy	23100283
GSE20129	atherosclerosis	21521779
GSE20680	coronary artery disease	21443790; 23210427
GSE20681	coronary artery disease	21443790; 23210427
GSE21610	heart failure	20460602
GSE22255	stroke	22453632
GSE2240	cardiac arrhythmias	15817885; 15877233
GSE23746	arteriosclerosis	n/a
GSE24752	hypertension	21369372
GSE24818	cardiovascular disease	n/a
GSE26887	heart failure	22427379
GSE27034	peripheral arterial disease	22409835
GSE28345	hypertension	22042811
GSE28360	hypertension	22042811
GSE29819	arrhythmogenic right ventricular cardiomyopathy	22085907
GSE34198	acute myocardial infarction	n/a
GSE36761	tetralogy of Fallot	24459294
GSE36791	intracranial aneurysm	23512133
GSE42148	coronary artery disease	24674750
GSE42955	ischemic cardiomyopathy;dilated cardiomyopathy	24339868
GSE43292	thromboembolism	23660665
GSE57691	abdominal aortic aneurysm; Leriche syndrome	25944698
GSE60217	coronary artery disease	27595325
GSE64486	Kawasaki disease	26679344
GSE65705	myocardial infarction	26367242
GSE66360	acute myocardial infarction	n/a
GSE71613	cardiomyopathies	n/a
GSE76899	atrial fibrillation	n/a
GSE9820	coronary artery disease	19059264
GSE17582	cardiorenal syndrome	22957013

Cardiovascular disease-related datasets used in the current study were extracted from OmicSoft DiseaseLand database, which contains datasets retrieved from a variety of public projects including GEO (Gene Expression Omnibus), SRA (Sequence Read Archive), ArrayExpress, and dbGAP (The Database of Genotypes and Phenotypes).

Supplementary Table S5. Downregulation of FGF19 in patients with cardiovascular disease

ID	Comparison	Fold Change [Log2]	P value	Platform Name	Sample Source [Disease]	Sample Source [Control]	Sample Size
GSE26887.GPL6244	Type 2 diabetes mellitus;heart failure vs normal control	−0.4102	0.0034	Affymetrix.HuGene-1_0-st-v1	ventricular myocardium	ventricular myocardium	11
GSE26887.GPL6244	Heart failure vs normal control	−0.3226	0.0082	Affymetrix.HuGene-1_0-st-v1	ventricular myocardium	ventricular myocardium	16
GSE43292.GPL6244	Atherosclerotic vs non-atherosclerotic	−0.1193	0.0085	Affymetrix.HuGene-1_0-st-v1	carotid atherosclerotic plaque	carotid artery	63
GSE1145.GPL570	Idiopathic cardiomyopathy vs normal control	0.2271	0.0104	Affymetrix.HG-U133_Plus_2	heart	heart	38
GSE57691.GPL10558	Leriche syndrome vs normal control	−0.2012	0.022	Illumina.HumanHT-12_V4_0_R1_15002873_B	aorta	aorta	19
GSE42955.GPL6244	Dilated cardiomyopathy vs normal control	−0.2729	0.041	Affymetrix.HuGene-1_0-st-v1	heart ventricles	heart ventricles	17

FGF19 RNA levels in patients with cardiovascular disease (a list of datasets is provided in Supplementary Table S4) were examined and compared using ArrayStudio software version 10.0 from OmicSoft. Negative Log2 fold change values indicate downregulation of FGF19 in subjects with disease relative to normal control subjects. Positive Log2 fold change values indicate upregulation of FGF19. Only comparisons with significant difference ($P<0.05$) are shown.

Supplementary Table S6. Baseline participants' characteristics in a double-blind, placebo-controlled trial of NGM282 in healthy human subjects

	Placebo (n=17)	NGM282 3 mg (n=9)
Age, years (SD)	28.8 (7.6)	29.8 (8.0)
Male, n (%)	17 (100)	9 (100)
Weight, kg (SD)	90.1 (10.5)	92.1 (8.0)
Height, cm (SD)	179.9 (4.6)	181.3 (5.8)
BMI, kg/m2 (SD)	27.8 (3.0)	28.0 (1.8)
Lipids, mean (SD)		
Cholesterol (mg/dL)	186.1 (37.2)	193.5 (33.7)
HDL-C (mg/dL)	52.2 (9.3)	50.3 (8.1)
LDL-C (mg/dL)	113.0 (36.0)	121.1 (32.1)
Triglycerides (mg/dL)	111.6 (42.5)	111.6 (28.4)

Shown are mean (SD) or n (%). BMI, body mass index; HDL-C, high-density lipoprotein cholesterol; LDL-C, low-density lipoprotein cholesterol; SD, standard deviation.

Supplementary Table S7. Change from baseline to day 7 in serum lipids in participants treated with NGM282

	Change From Baseline to Day 7, LS Mean (SE)		LS Mean Difference (95% CI)	
	**Placebo (n=17)**	**NGM282 (n=9)**	**NGM282 vs. Placebo**	**P**
Cholesterol (mg/dL)	−8.1 (3.9)	40.6 (5.4)	48.8 (31.0, 66.6)	<0.001
HDL-C (mg/dL)	−5.4 (1.2)	8.1 (1.9)	13.5 (7.7, 19.7)	<0.001
LDL-C (mg/dL)	−3.1 (4.3)	41.4 (5.8)	44.5 (25.5, 63.9)	<0.001
Triglycerides (mg/dL)	−8.9 (7.1)	−53.2 (9.7)	−44.3 (−76.2, −12.4)	0.003

CI, confidence interval; HDL-C, high-density lipoprotein cholesterol; LDL-C, low-density lipoprotein cholesterol; LS, least-squares; SE, standard error.

SAS (version 9.4) was used for all analyses. The difference between NGM282 and placebo groups was analyzed using analysis of covariance (ANCOVA) model with treatment group as the factor and baseline values of the outcome as cofactor. All statistical analyses were carried out using two-sided tests at the 5% level of significance. Least-squares means, difference in least-squares means, 95% confidence intervals (CI) for the difference and corresponding P-values were presented.

Supplementary Table S8. Percent change from baseline to day 7 in serum lipids in participants treated with NGM282

	Percent Change From Baseline to Day 7, LS Mean (SE)		LS Mean Difference (95% CI)	
	**Placebo (n=17)**	**NGM282 (n=9)**	**NGM282 vs. Placebo**	**P**
Cholesterol (mg/dL)	−3.7 (2.0)	21.0 (2.8)	24.7 (15.5, 33.9)	<0.001
HDL-C (mg/dL)	−9.8 (2.4)	16.7 (3.3)	26.5 (15.6, 37.5)	<0.001
LDL-C (mg/dL)	−0.5 (6.7)	37.2 (9.3)	37.7 (6.8, 68.5)	0.01
Triglycerides (mg/dL)	−5.1 (5.9)	−42.8 (8.1)	−37.6 (−64.3, −11.0)	0.002

CI, confidence interval; HDL-C, high-density lipoprotein cholesterol; LDL-C, low-density lipoprotein cholesterol; LS, least-squares; SE, standard error.

SAS (version 9.4) was used for all analyses. The difference between NGM282 and placebo groups was analyzed using analysis of covariance (ANCOVA) model with treatment group as the factor and baseline values of the outcome as cofactor. All statistical analyses were carried out using two-sided tests at the 5% level of significance. Least-squares means, difference in least-squares means, 95% confidence intervals (CI) for the difference and corresponding P-values were presented.

Supplementary Table S9. Clinical chemistry reagents used in the study

Reagent Name	Vendor	Catalog Number
Cholesterol (Gen.2)	Roche Diagnostics	Cat. No. 03039773 190<br>System-ID 07 6726 3
HDL-Cholesterol (plus 3rd generation)	Roche Diagnostics	Cat. No. 04399803 190<br>System-ID 07 6833 2
LDL-Cholesterol (plus 2nd generation)	Roche Diagnostics	Cat. No. 03038866 322<br>System-ID 07 6627 5
Aspartate Aminotransferase	Roche Diagnostics	Cat. No. 20764949 322<br>System-ID 07 6494 9
Calibrators and controls:		
Calibrator f.a.s. Lipids	Roche Diagnostics	Cat. No. 12172623 160<br>System-ID 07 6570 8
Calibrator f.a.s.	Roche Diagnostics	Cat. No. 10759350 360<br>System-ID 07 3718 6
Precinorm L	Roche Diagnostics	Cat. No. 10781827 122<br>System-ID 07 9026 5
Precipath HDL/LDL-C	Roche Diagnostics	Cat. No. 11778552 122<br>System-ID 07 9028 1
Precinorm U plus	Roche Diagnostics	Cat. No. 12149435 122<br>System-ID 07 7999 7
Precipath U plus	Roche Diagnostics	Cat. No. 12149443 122<br>System-ID 07 8000 6

Reagents used to measure levels of total cholesterol, HDL-C, LDL-C, and aspartate aminotransferase in mouse serum samples on COBAS INTEGRA 400-Plus Clinical Analyzer (Roche Diagnostics).

Supplementary Table S10. Primers for quantitative real-time PCR analysis

Gene Symbol	Vendor	Catalog Number	Assay ID	Label
Abca1	Life Technologies	4331182	Mm00442646_m1	FAM-MGB
Abcg1	Life Technologies	4331182	Mm00437390_m1	FAM-MGB
Abcg5	Life Technologies	4331182	Mm00446241_m1	FAM-MGB
Abcg8	Life Technologies	4331182	Mm00445980_m1	FAM-MGB
Apoa1	Life Technologies	4331182	Mm00437569_m1	FAM-MGB
Cyp7a1	Life Technologies	4331182	Mm00484150_m1	FAM-MGB
Fasn	Life Technologies	4331182	Mm00662319_m1	FAM-MGB
Lpcat3	Life Technologies	4331182	Mm00520147_m1	FAM-MGB
Scap	Life Technologies	4331182	Mm01250176_m1	FAM-MGB
Scarb1	Life Technologies	4331182	Mm00450234_m1	FAM-MGB
Scd1	Life Technologies	4331182	Mm00772290_m1	FAM-MGB
Srebf1	Life Technologies	4331182	Mm00550338_m1	FAM-MGB
Srebf2	Life Technologies	4331182	Mm01306292_m1	FAM-MGB

Taqman Gene Expression assays used for quantitative reverse transcription PCR on RNA extracted from mouse livers and ileums.
